# Supplementary figures and images for: Cholinergic-like neurons carrying PSEN1 E280A mutation from familial Alzheimer’s disease reveal intraneuronal sAPPβ fragments accumulation, hyperphosphorylation of TAU, oxidative stress, apoptosis and Ca2+ dysregulation: Therapeutic implications
Source: PLoS One. 2020 May 21;15(5):e0221669. doi: 10.1371/journal.pone.0221669 (PMC7241743; doi:10.1371/journal.pone.0221669)

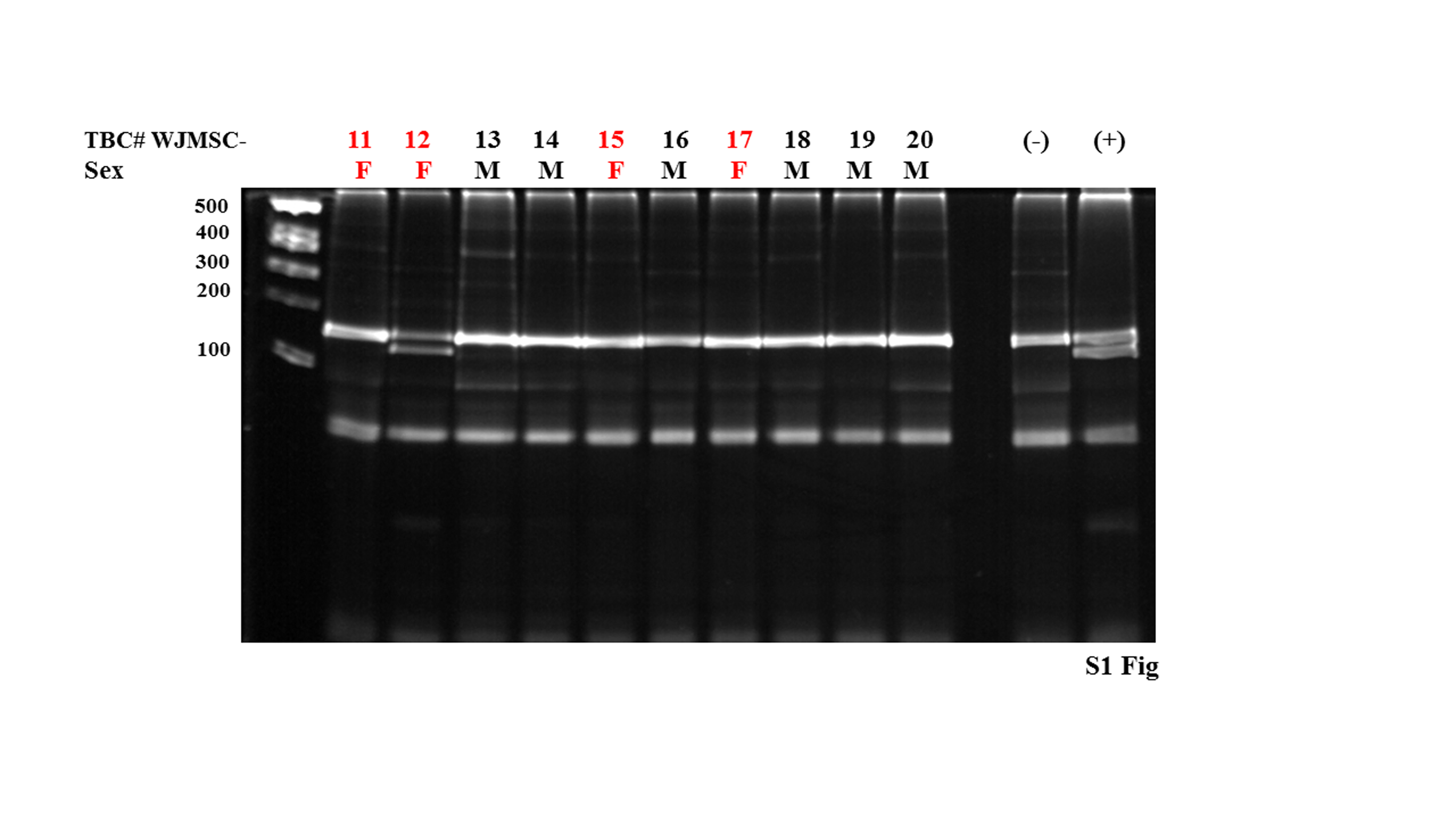

Supplement: S1 Fig — The PSEN1 E280A mutation was detected as described in Materials and Methods section. According to different mobility electrophoretic patterns, samples were classified as wild-type (WT) or mutant PSEN1 E280A when compared to PSEN1 E280A carrier (positive case NB code#18233) or wild type PSEN1 genotype (NB code#18574). Abbreviations: TBC# = tissue bank code number; F = female, M = male; (+) = positive; (-) = negative; numbers on the left are molecular size markers expressed in bp. (TIF) [file pone.0221669.s001.tif]

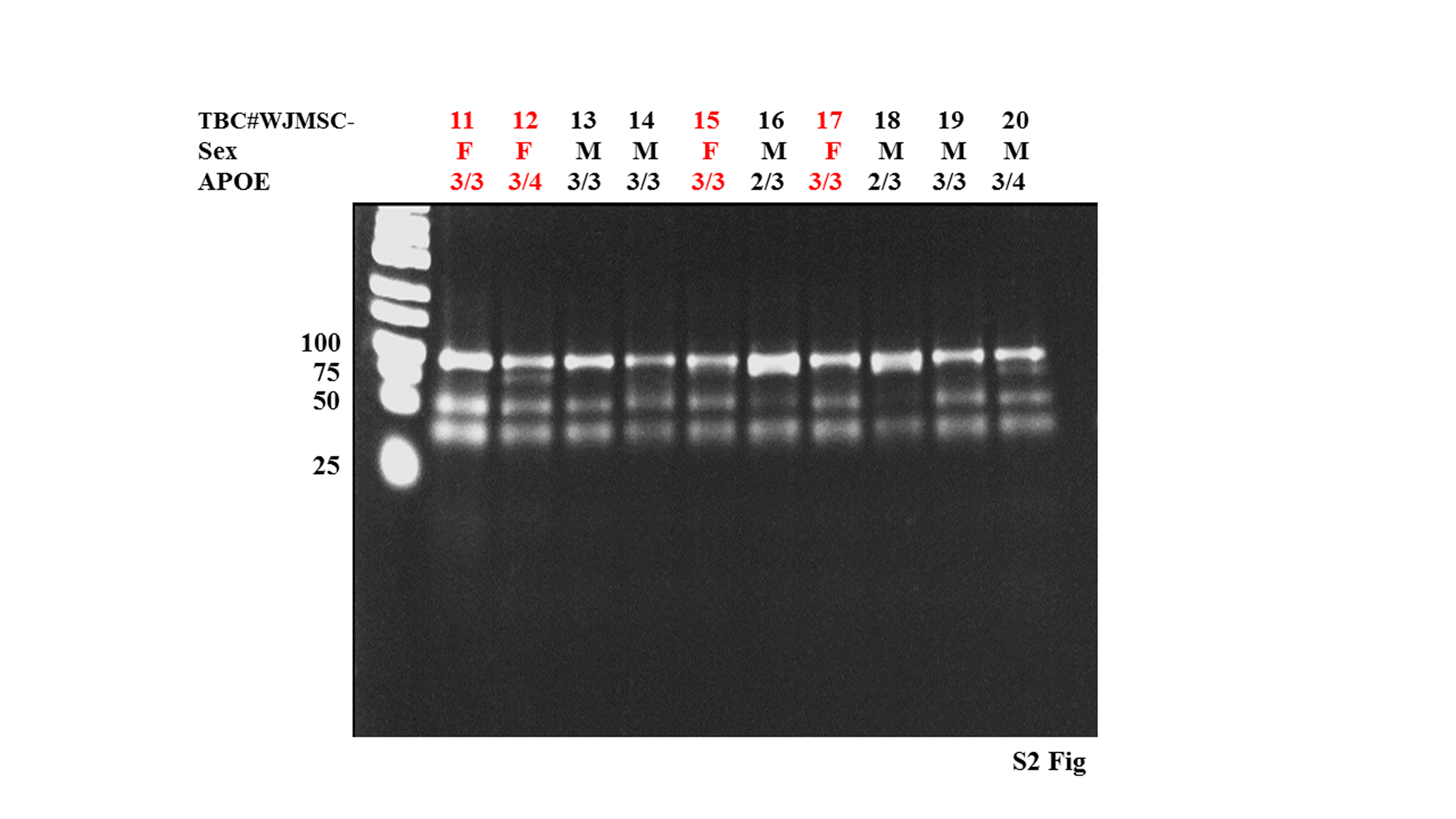

Supplement: S2 Fig — Electrophoretic separation of HhaI fragments after gene amplification of DNA from WT PSEN1 and PSEN1 E280A WJ-MSCs. The DNA band pattern was determined according to ref. [29]. Numbers on the left are molecular size markers expressed in bp. Abbreviations: TBC# = tissue bank code number; F = female, M = male. (TIF) [file pone.0221669.s002.tif]

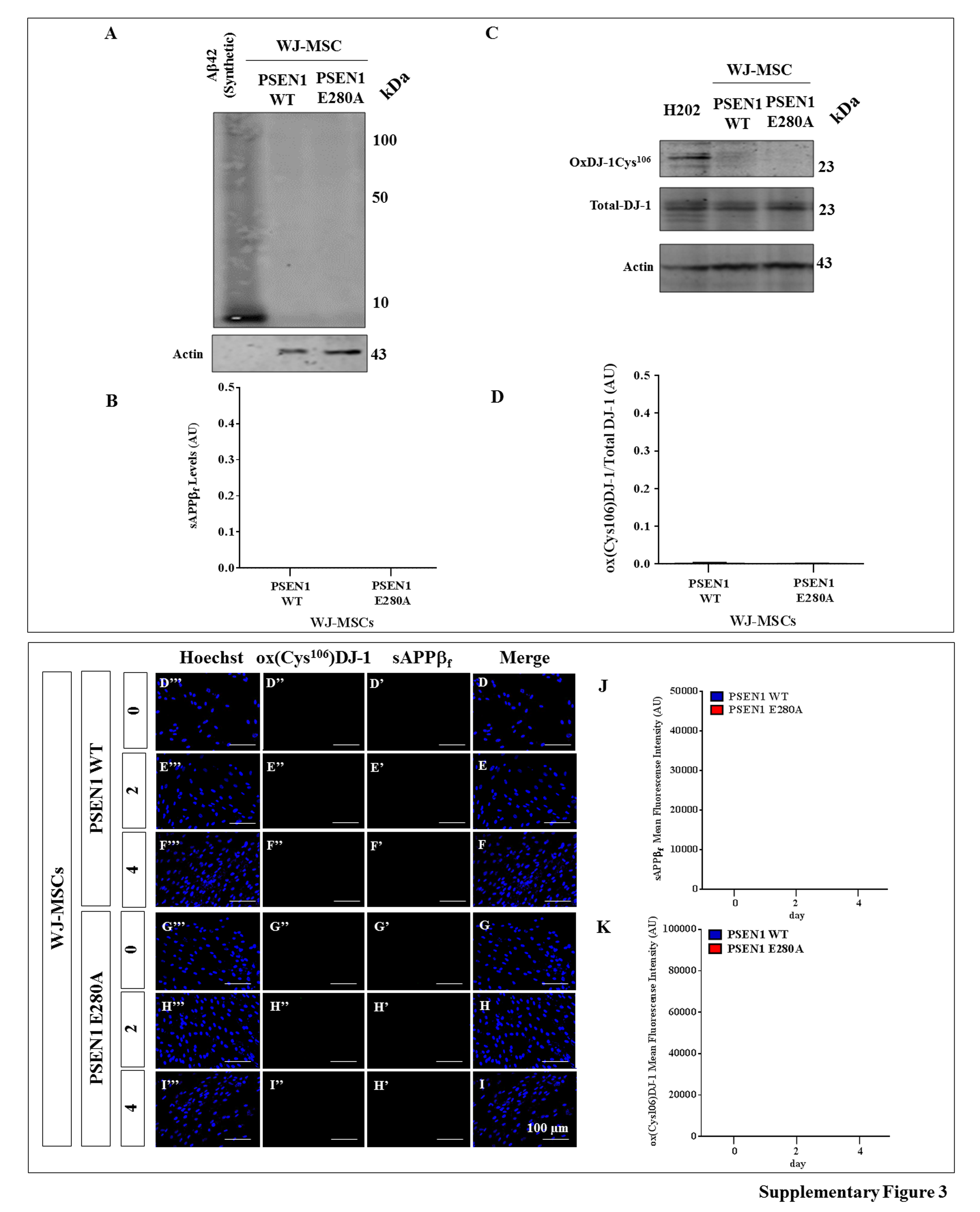

Supplement: S3 Fig — WT PSEN1 and PSEN1 E280A WJ-MSCs were cultured in MCm for 7 days, and then cultured for 4 additional days in regular culture medium (RCm). Then, the proteins in the extracts and control extracts were blotted with primary antibodies against Aβ42, oxDJ-1Cys106 and actin proteins. The intensities of the western blot bands shown in (A) were measured (B, C) by an infrared imaging system (Odyssey, LI-COR), and the intensity was normalized to that of actin. Control lysates were included to validate the results. Additionally, after 0, 2 and 4 days, WJ-MSCs were double stained as indicated in the figure (D-I) with primary antibodies against Aβ42 (red; D’-I’) and oxDJ-1Cys106 (green; D’-I”). The nuclei were stained with Hoechst 33342 (blue; D”‘-I”‘). (J) Quantification of Aβ42 fluorescence intensity. (K) Quantification of oxDJ-1Cys106 fluorescence as MFI. Data are expressed as the mean ± SD; *p<0.05; **p<0.01; ***p<0.001. The blots and figures represent 1 out of 3 independent experiments. Image magnification, 200x. (TIF) [file pone.0221669.s003.tif]

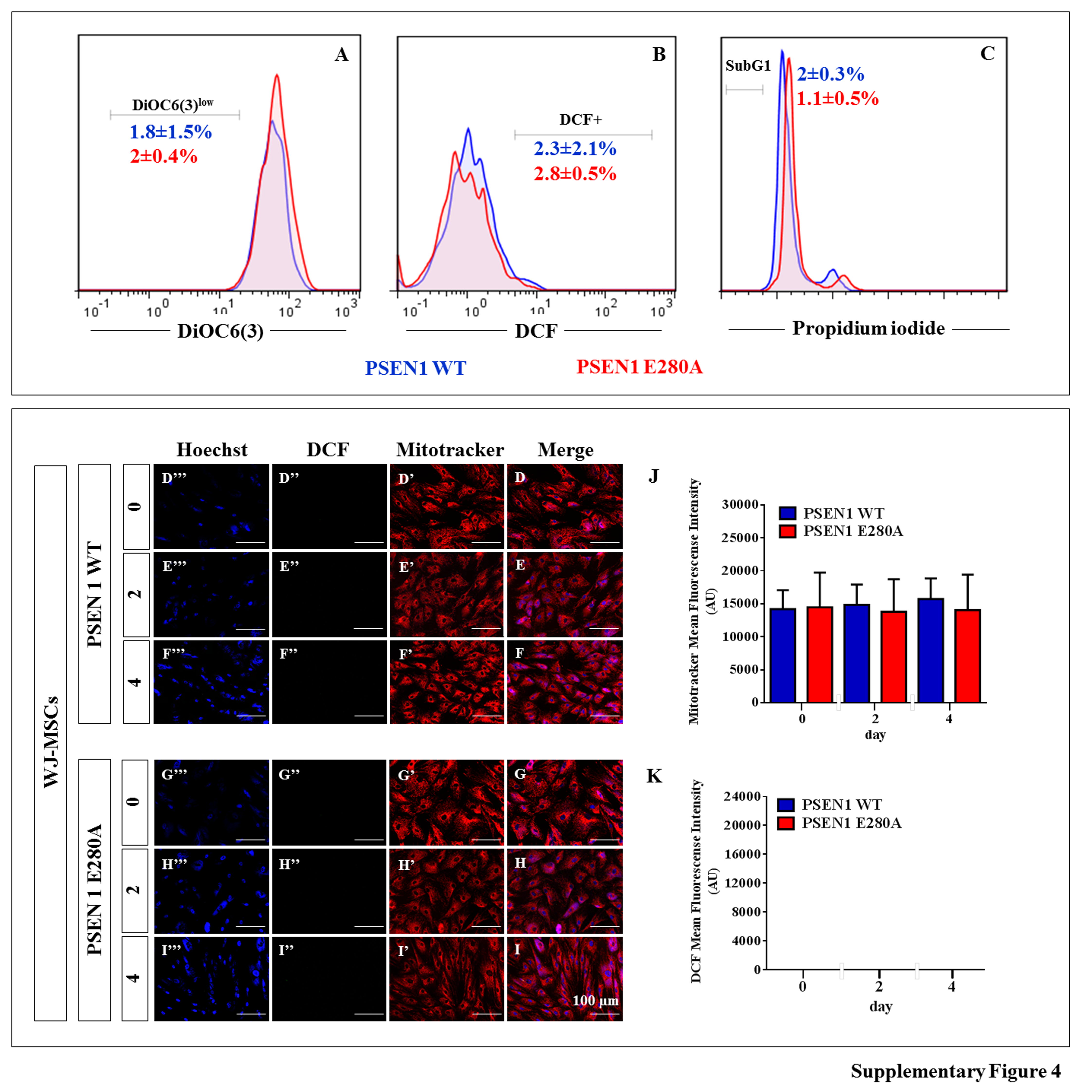

Supplement: S4 Fig — Representative histograms showing DiOC6(3)low (A), DCF+ (B) and SubG1 (C) populations from WT PSEN1 (blue) and PSEN1 E280A (Red) WJ-MSCs after 7 days in MCm plus 4 days in RCm. Representative MitoTracker (D’-I’), DCF (D”-I”), Hoechst (D”‘-I”‘) and merged (D-I) pictures of WT PSEN1 and PSEN1 E280A WJ-MSCs after 0, 2 and 4 days in RCm. (J) Quantification of MitoTracker fluorescence intensity. (K) Quantification of DCF fluorescence intensity. Data are expressed as the mean ± SD; *p<0.05; **p<0.01; ***p<0.001. The histograms and figures represent 1 out of 3 independent experiments. Image magnification, 200x. (TIF) [file pone.0221669.s004.tif]

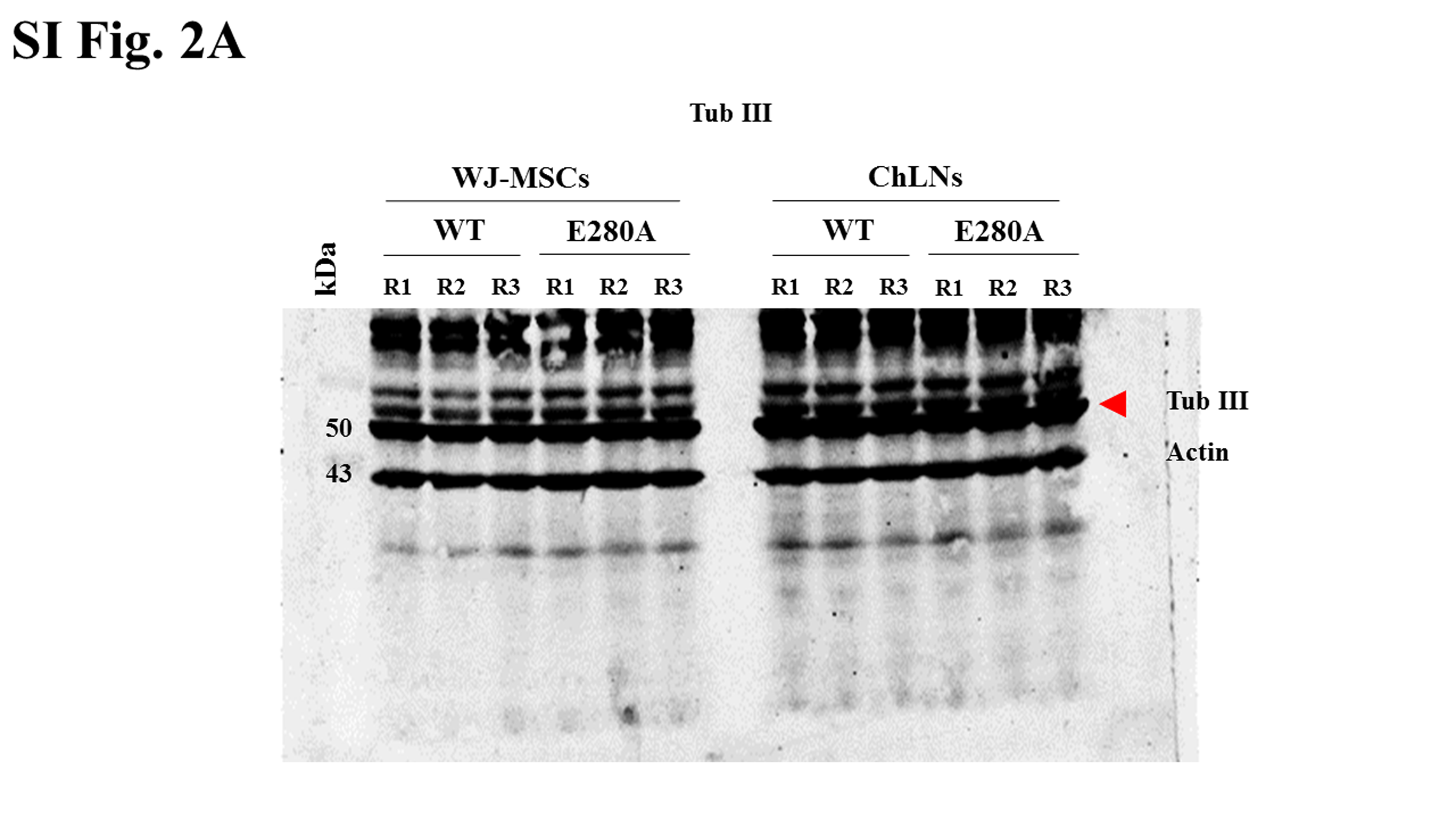

Supplement: S1 File — (ZIP) [file pone.0221669.s006.zip › 300dpi Support Info/S1 I.tif]

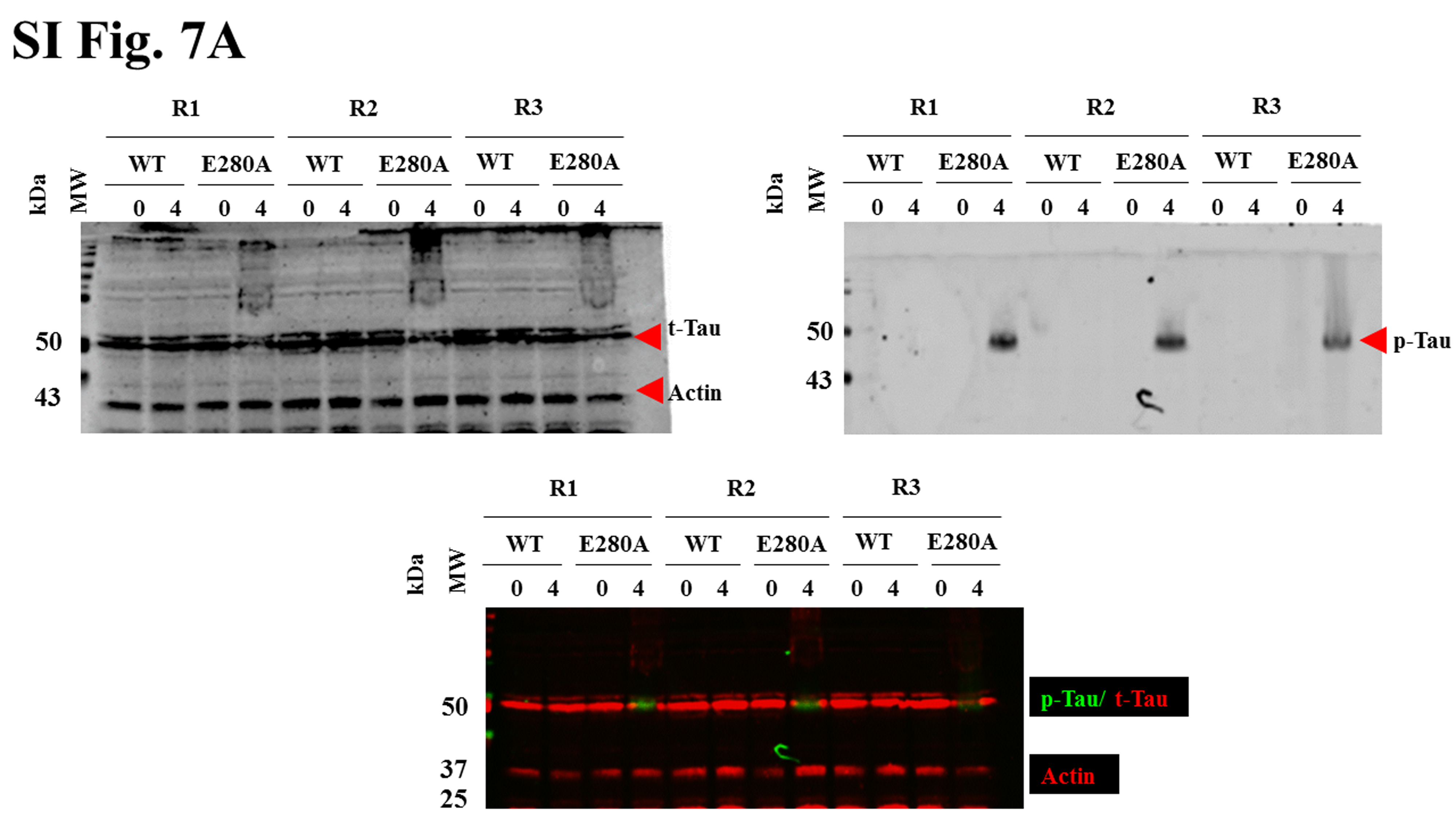

Supplement: S1 File — (ZIP) [file pone.0221669.s006.zip › 300dpi Support Info/S10 I.tif]

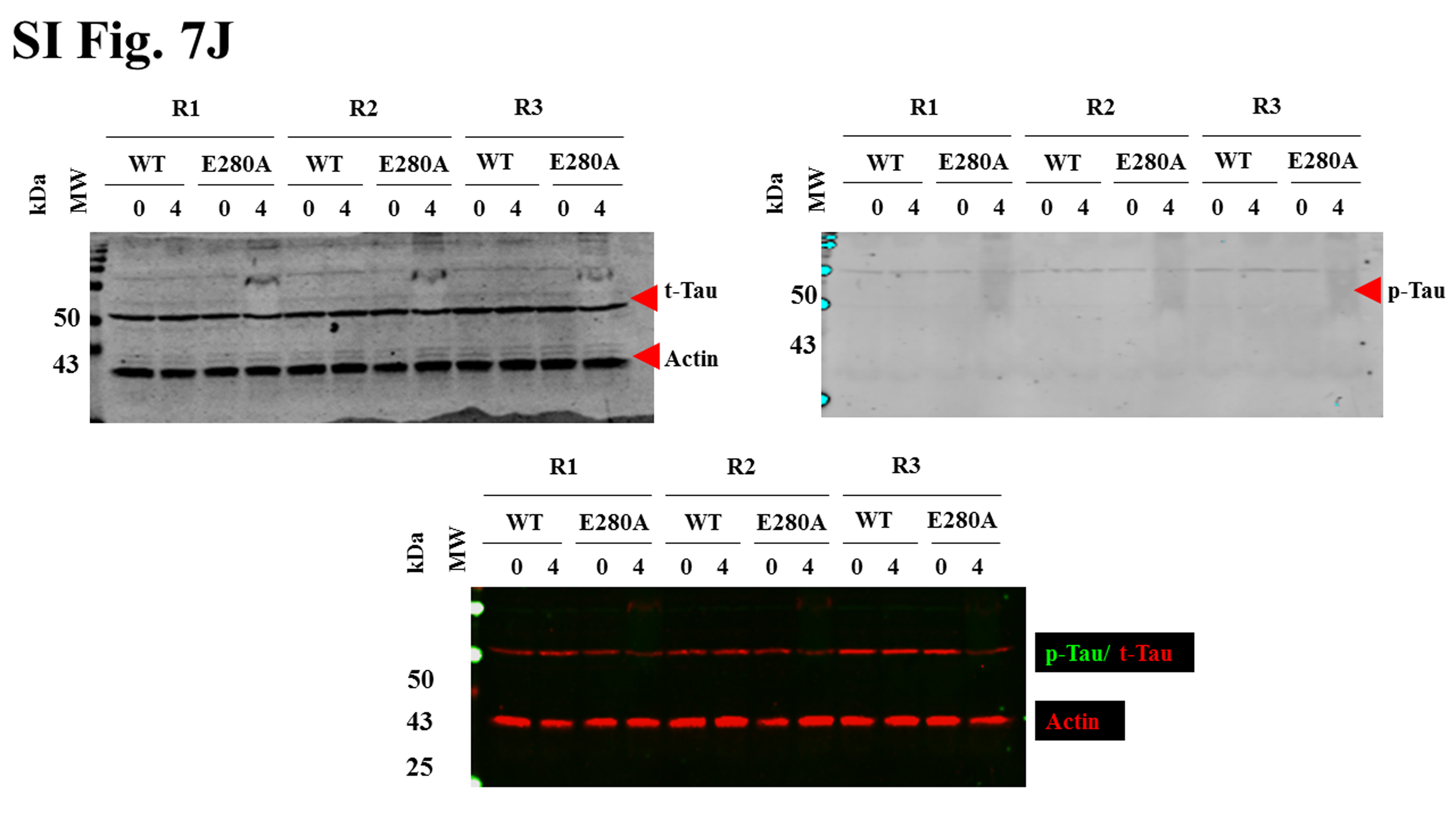

Supplement: S1 File — (ZIP) [file pone.0221669.s006.zip › 300dpi Support Info/S11 I.tif]

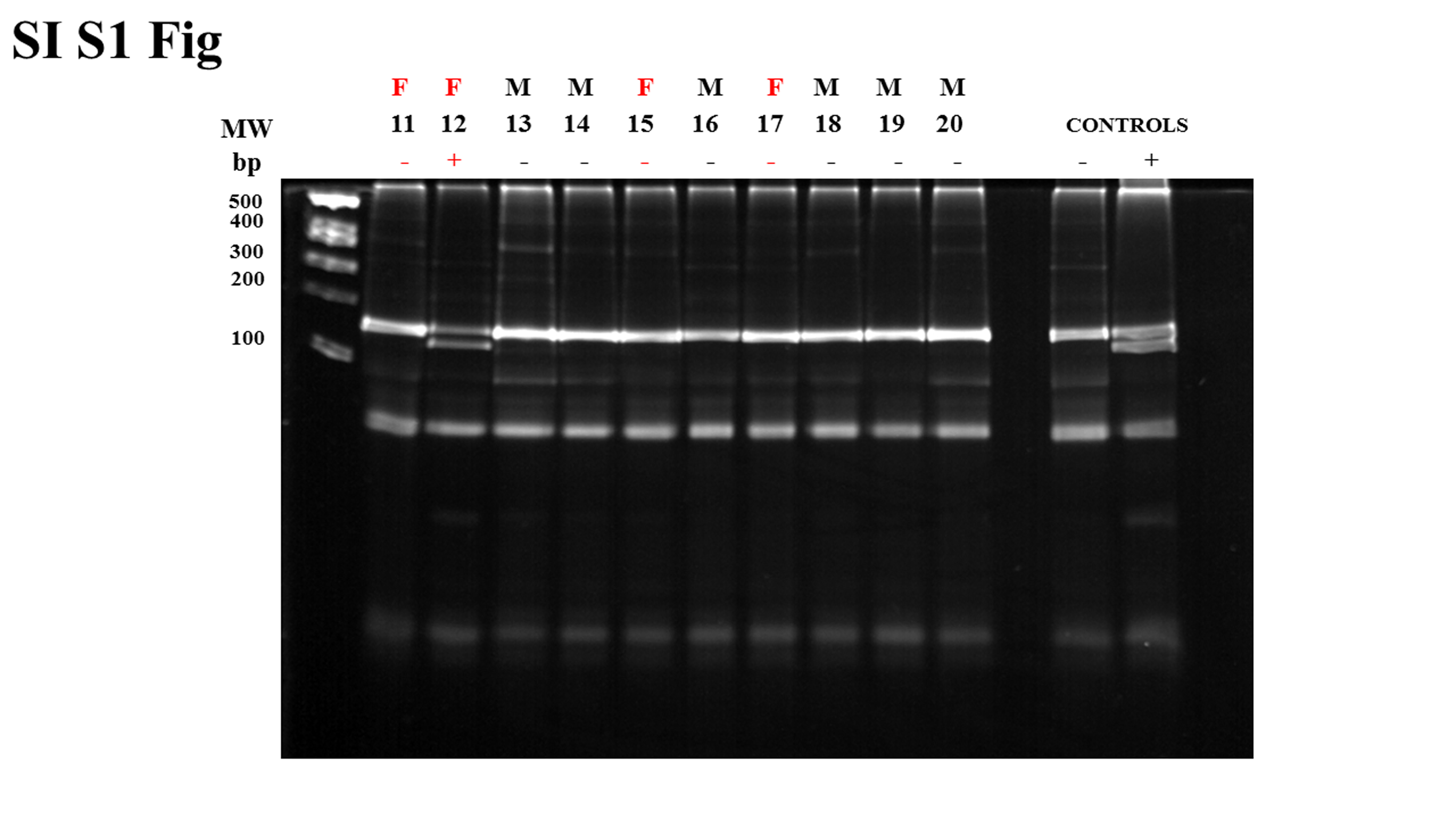

Supplement: S1 File — (ZIP) [file pone.0221669.s006.zip › 300dpi Support Info/S12 I.tif]

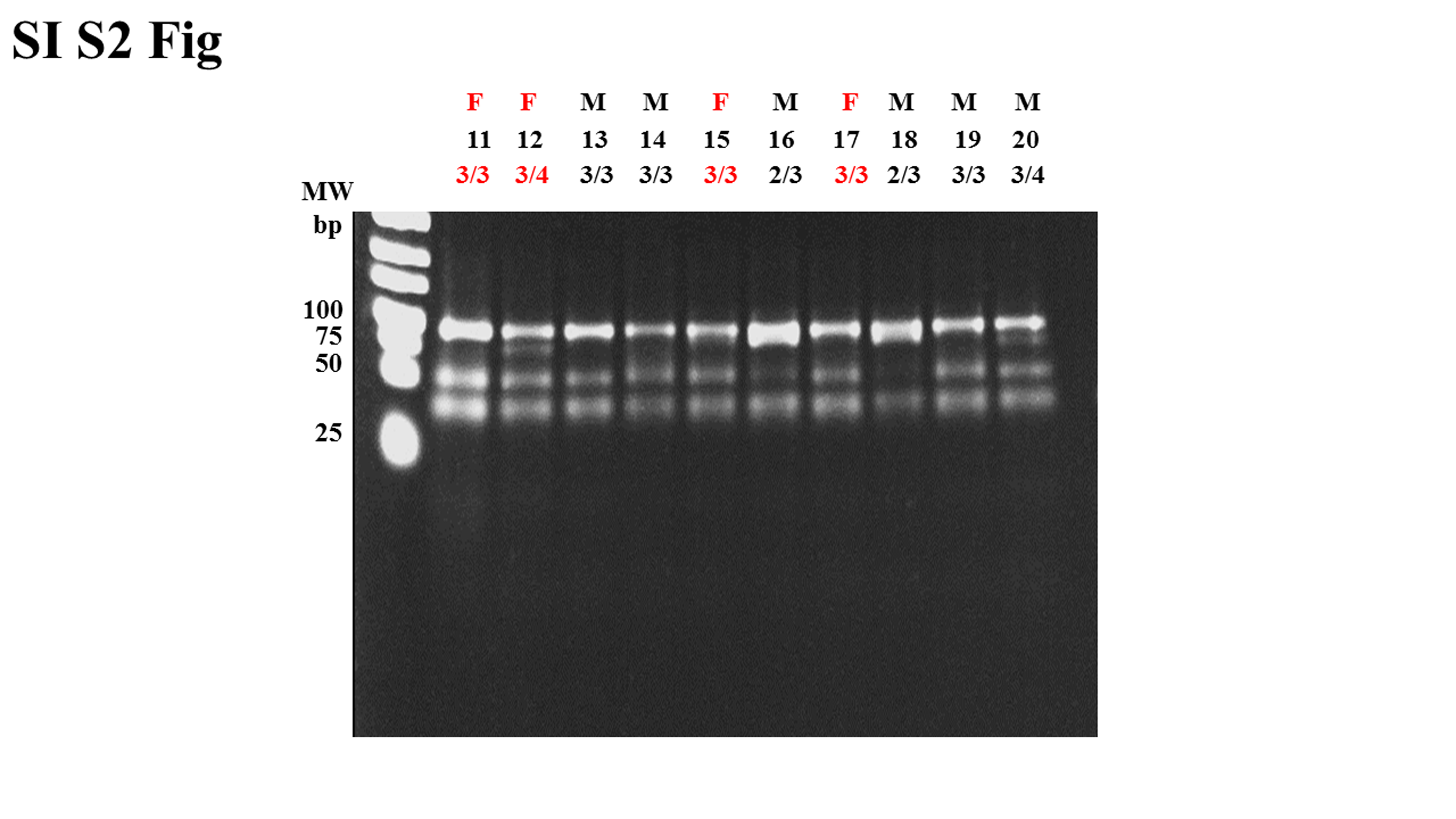

Supplement: S1 File — (ZIP) [file pone.0221669.s006.zip › 300dpi Support Info/S13 I.tif]

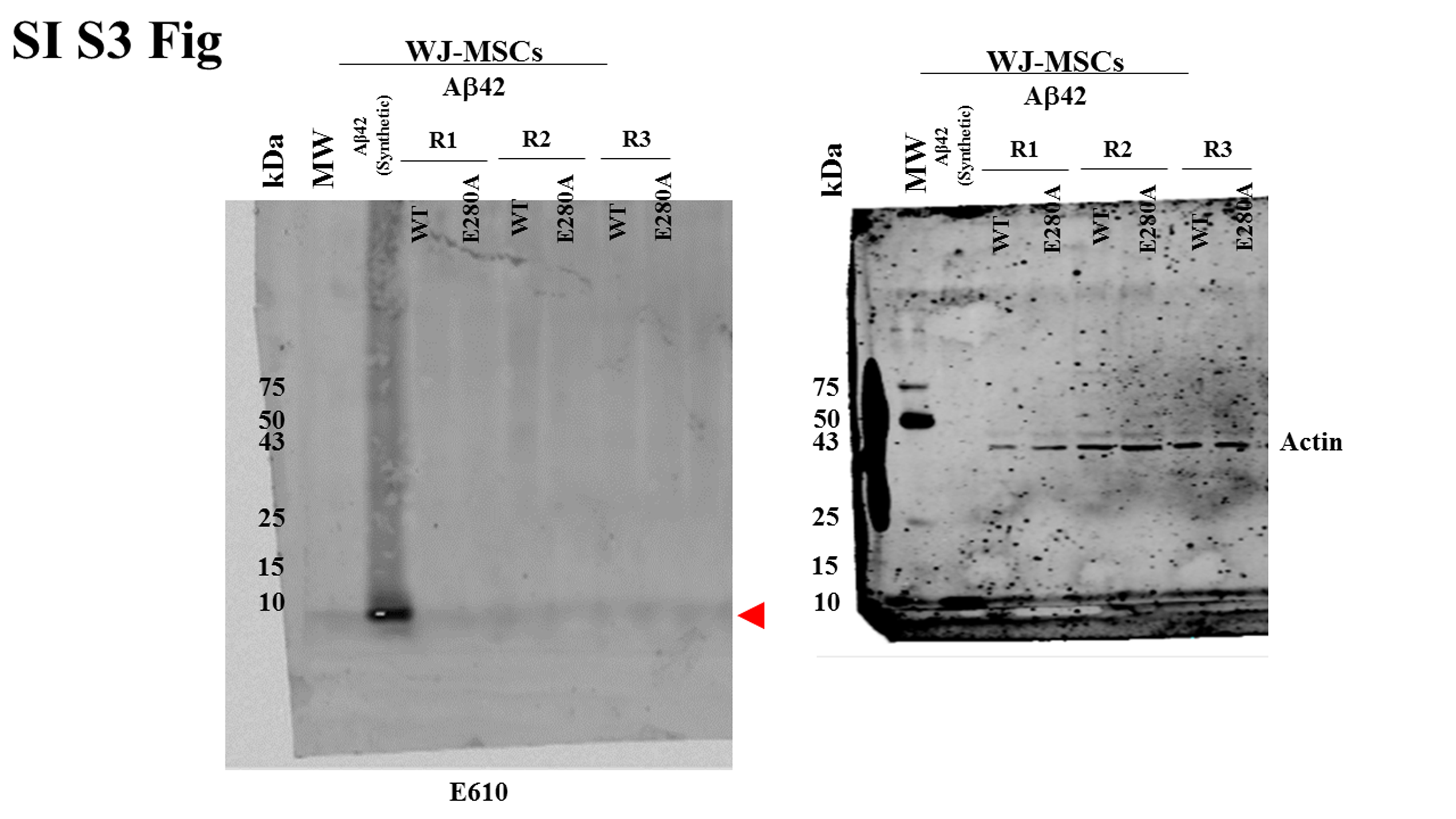

Supplement: S1 File — (ZIP) [file pone.0221669.s006.zip › 300dpi Support Info/S14 .tif]

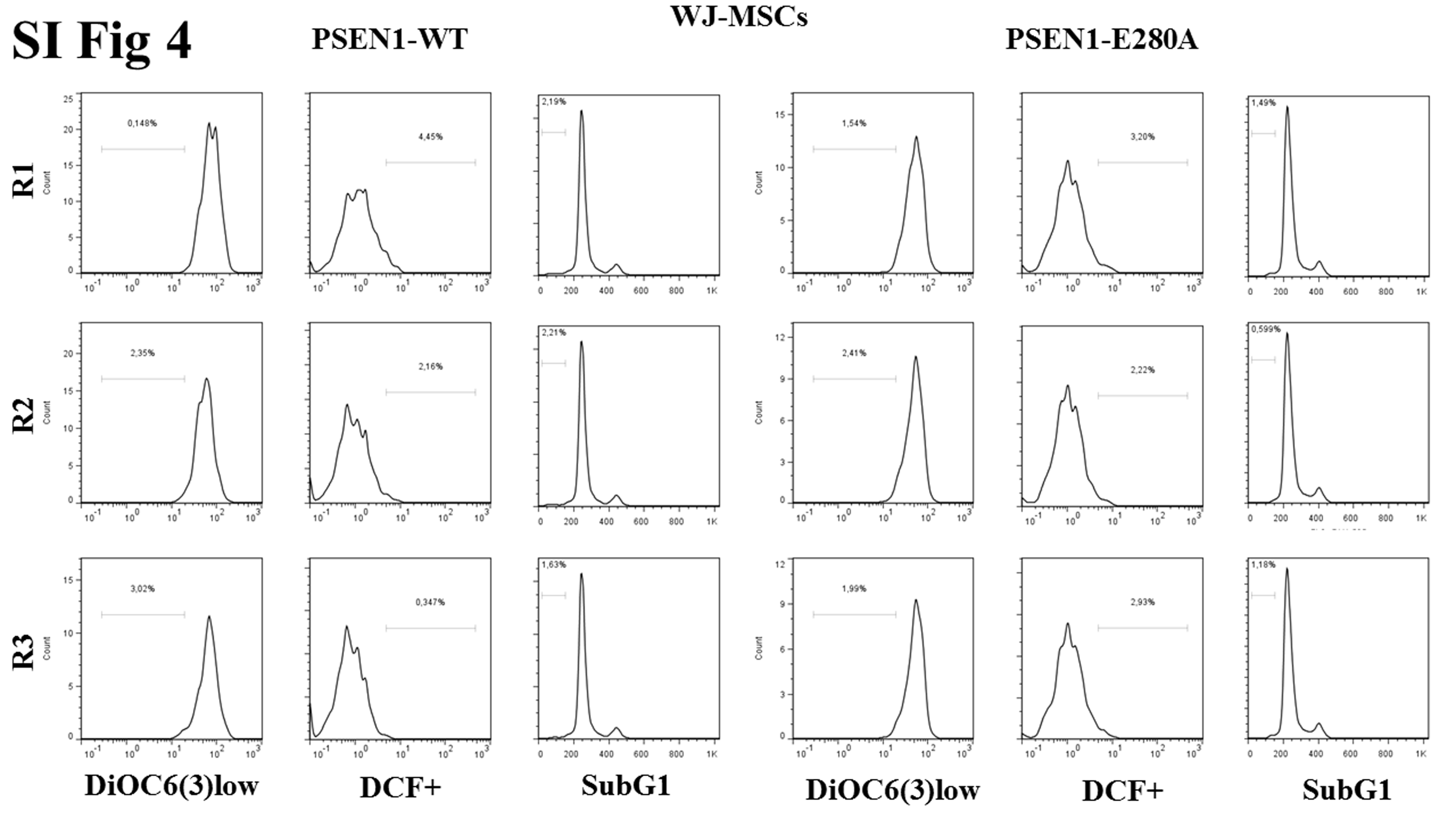

Supplement: S1 File — (ZIP) [file pone.0221669.s006.zip › 300dpi Support Info/S15 I.tif]

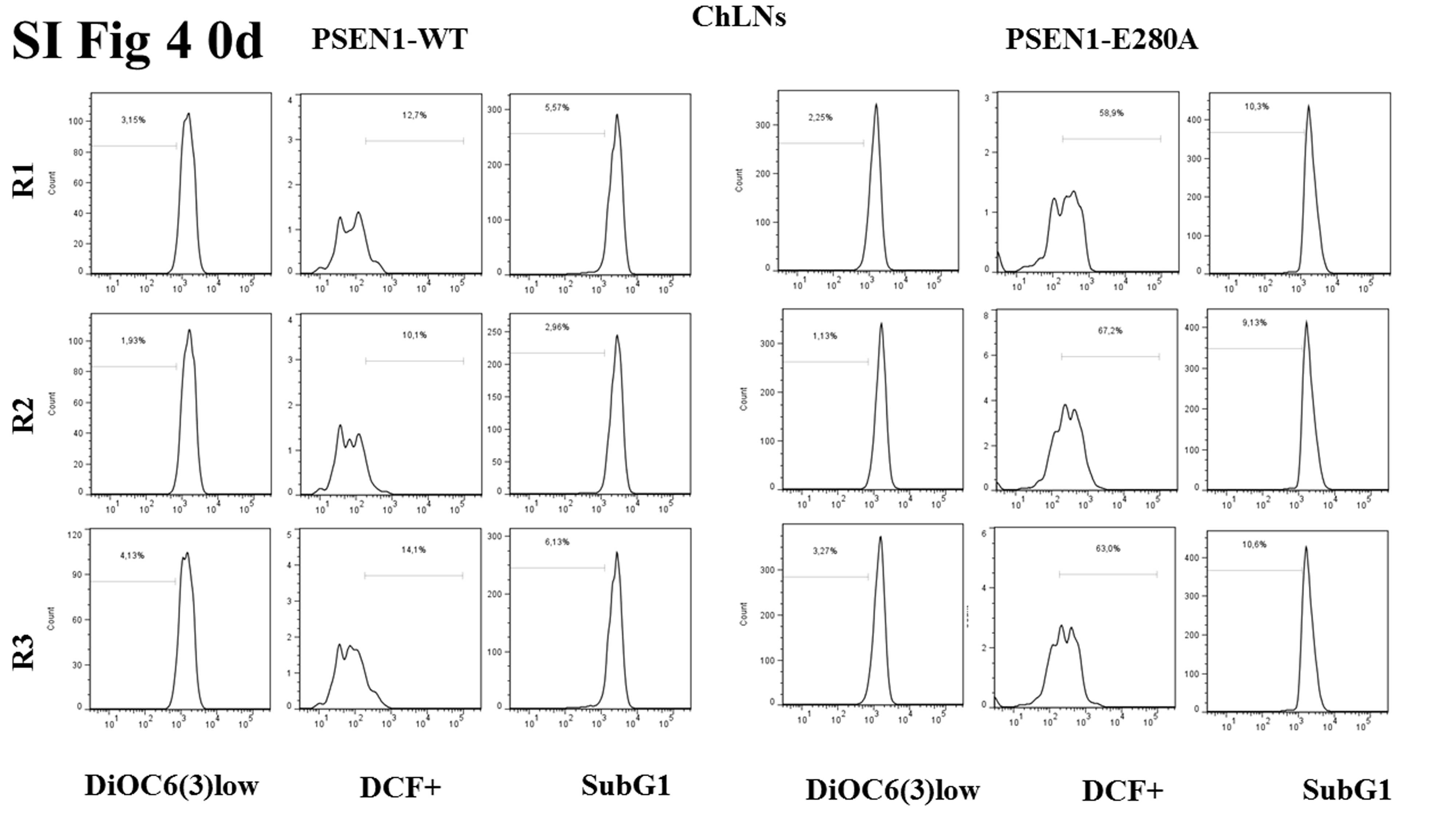

Supplement: S1 File — (ZIP) [file pone.0221669.s006.zip › 300dpi Support Info/S16 I.tif]

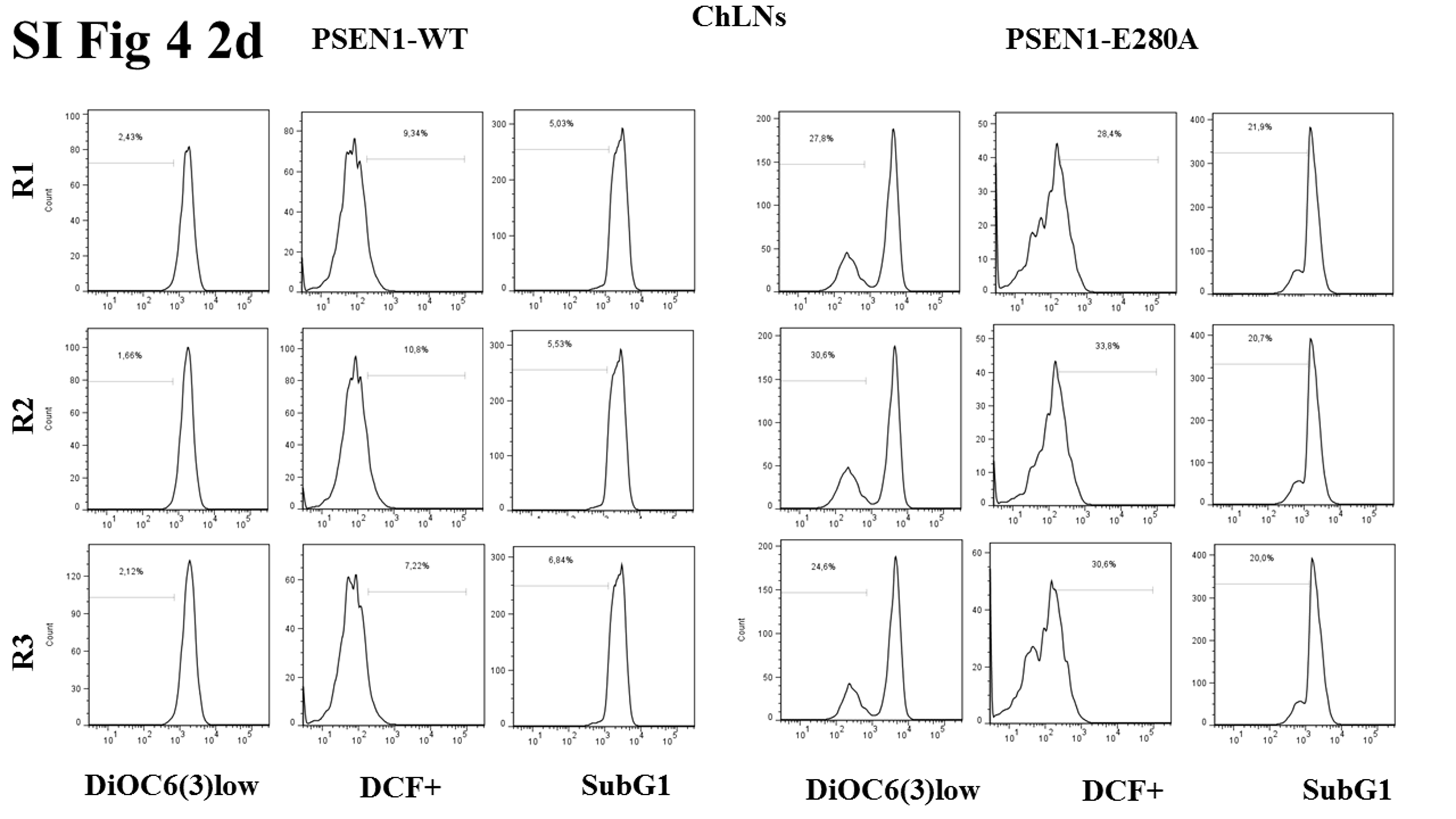

Supplement: S1 File — (ZIP) [file pone.0221669.s006.zip › 300dpi Support Info/S17 I.tif]

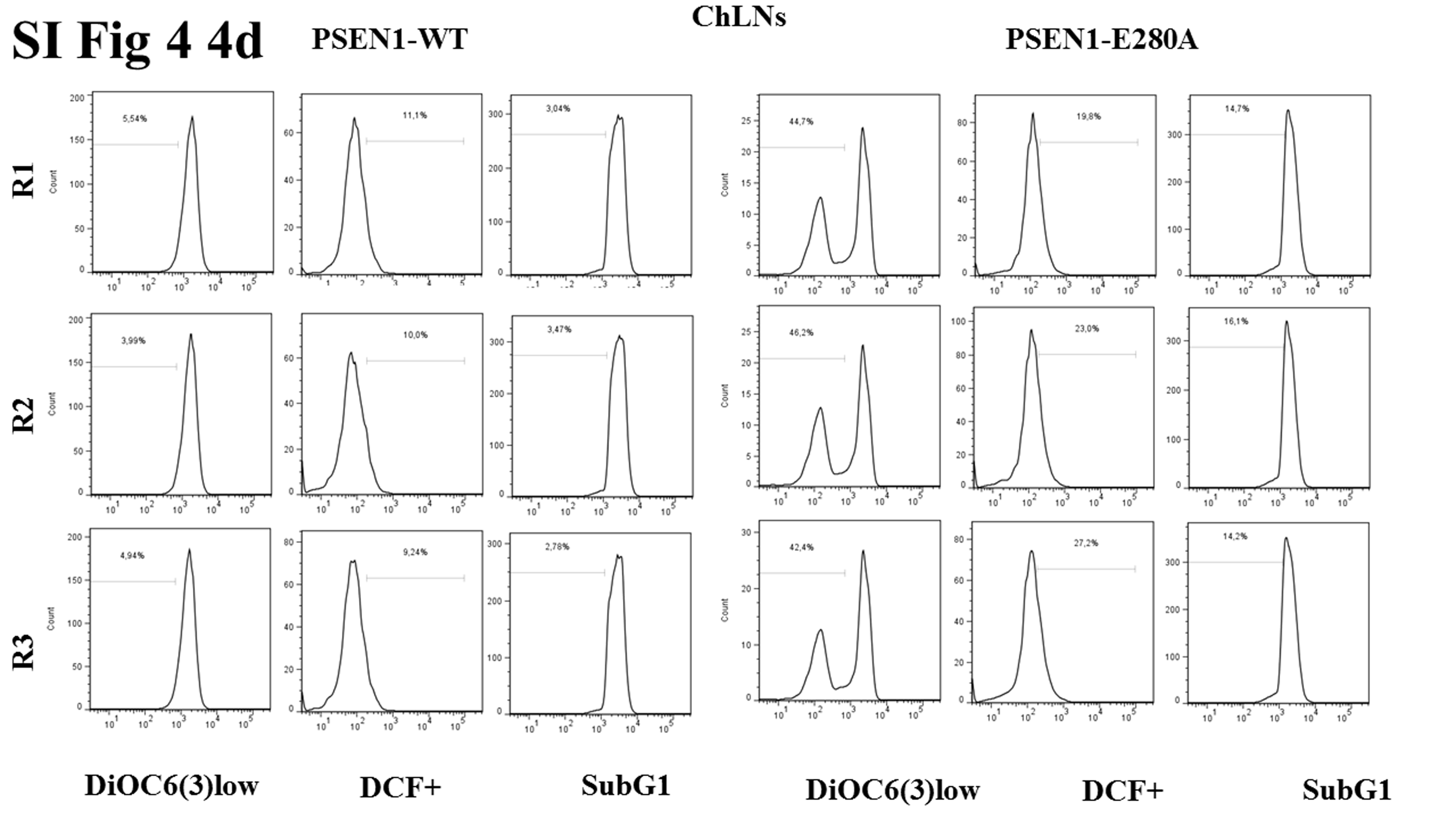

Supplement: S1 File — (ZIP) [file pone.0221669.s006.zip › 300dpi Support Info/S18 I.tif]

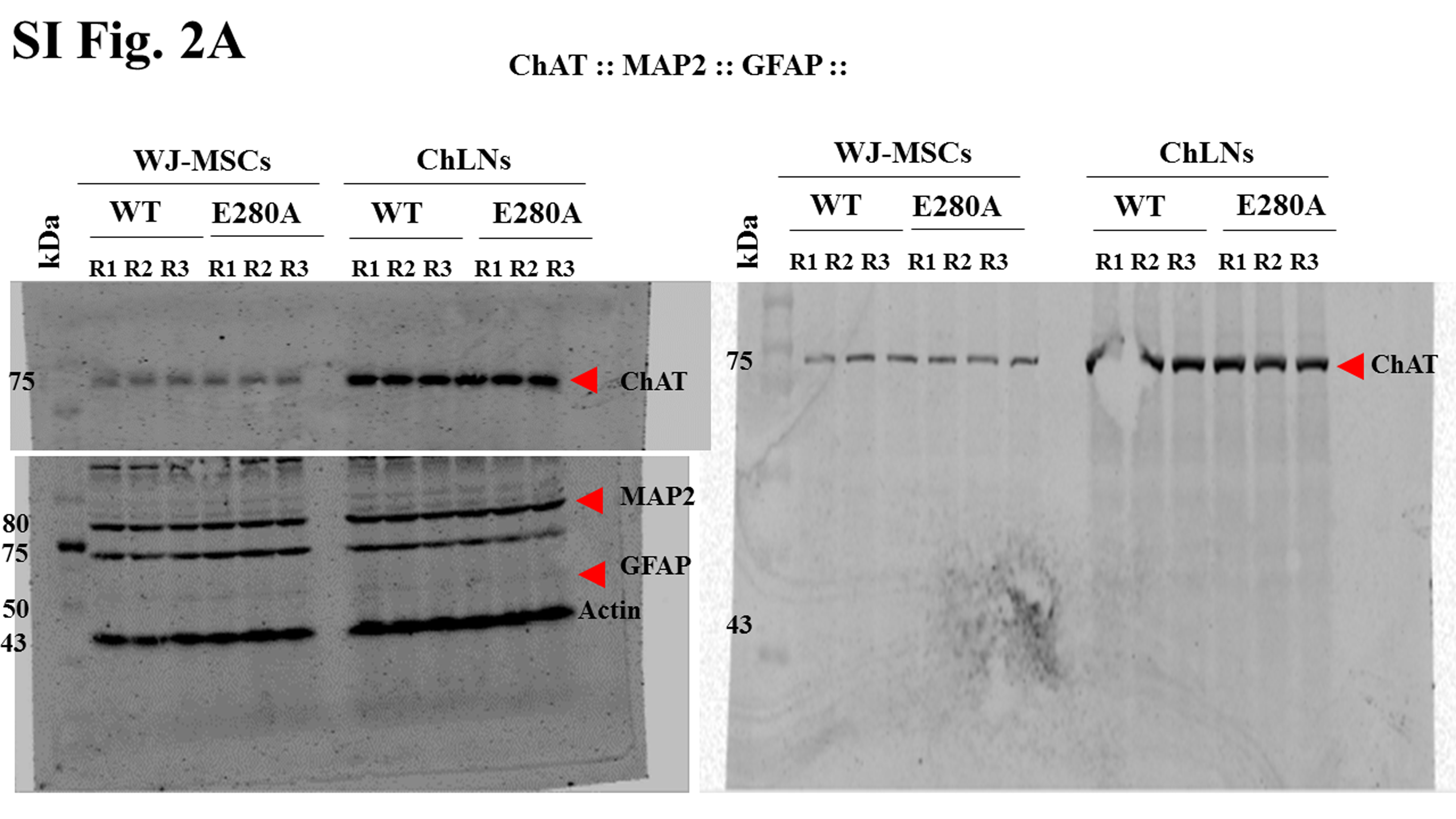

Supplement: S1 File — (ZIP) [file pone.0221669.s006.zip › 300dpi Support Info/S2 I.tif]

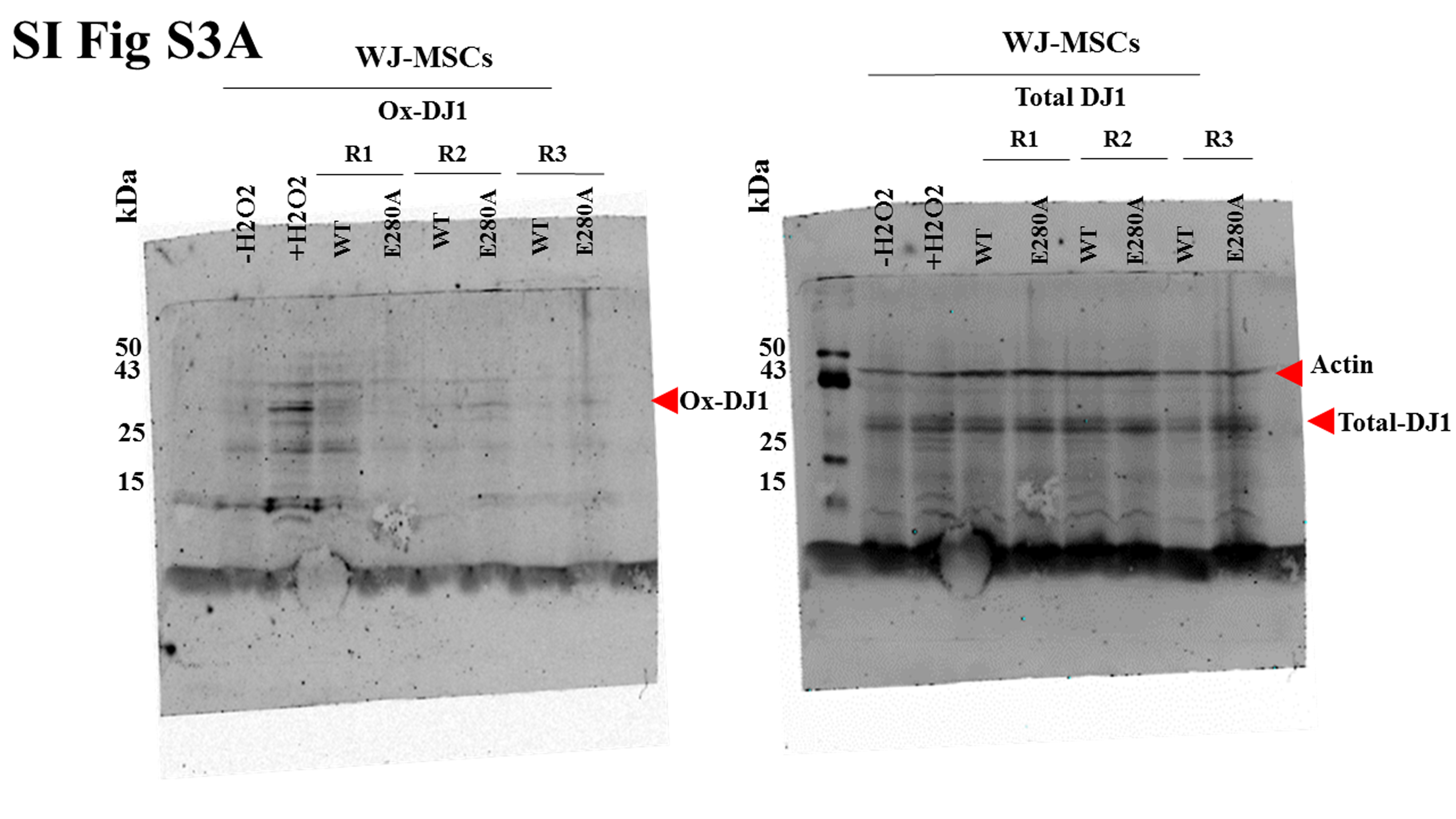

Supplement: S1 File — (ZIP) [file pone.0221669.s006.zip › 300dpi Support Info/S3 I.tif]

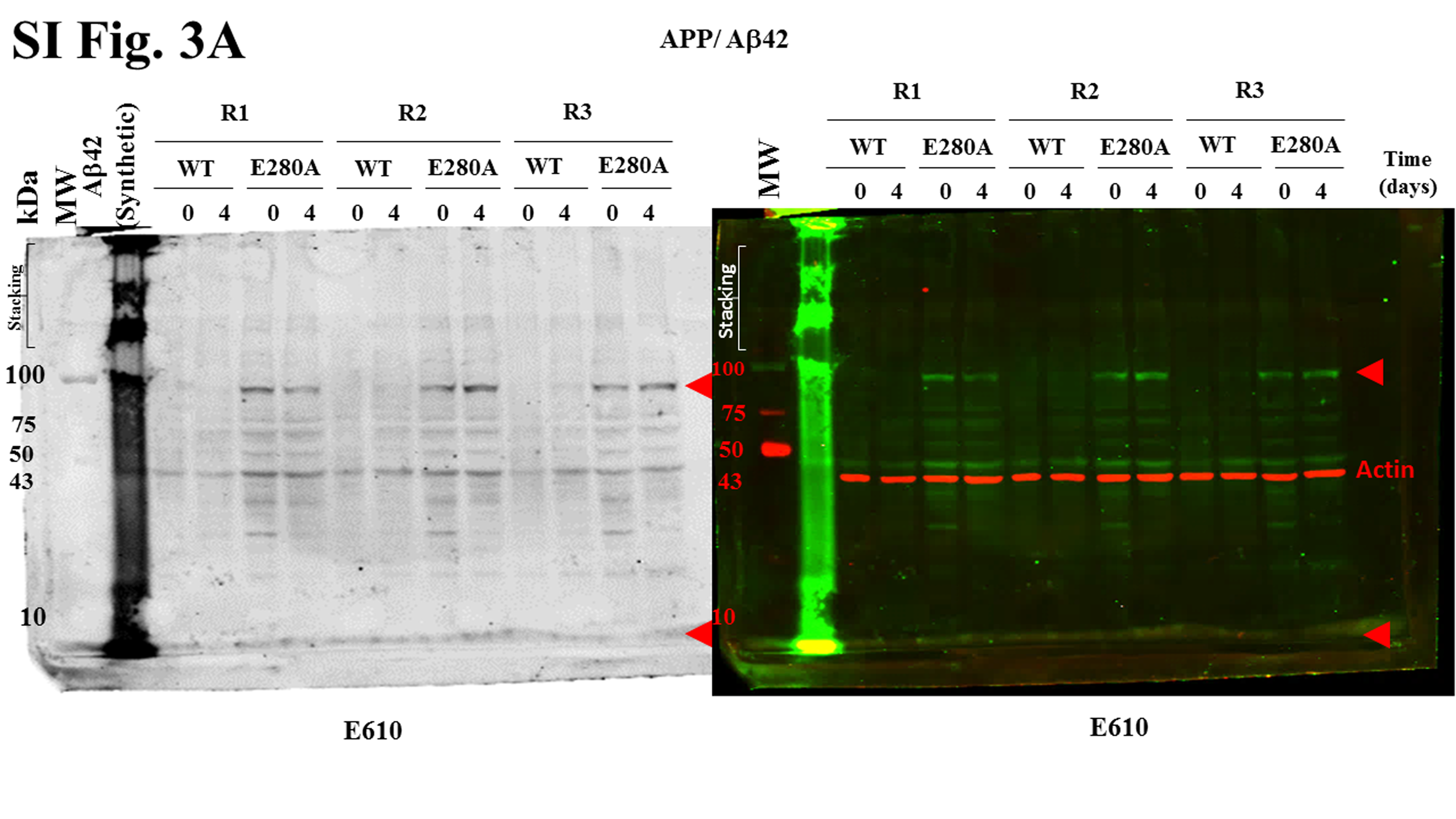

Supplement: S1 File — (ZIP) [file pone.0221669.s006.zip › 300dpi Support Info/S4 I.tif]

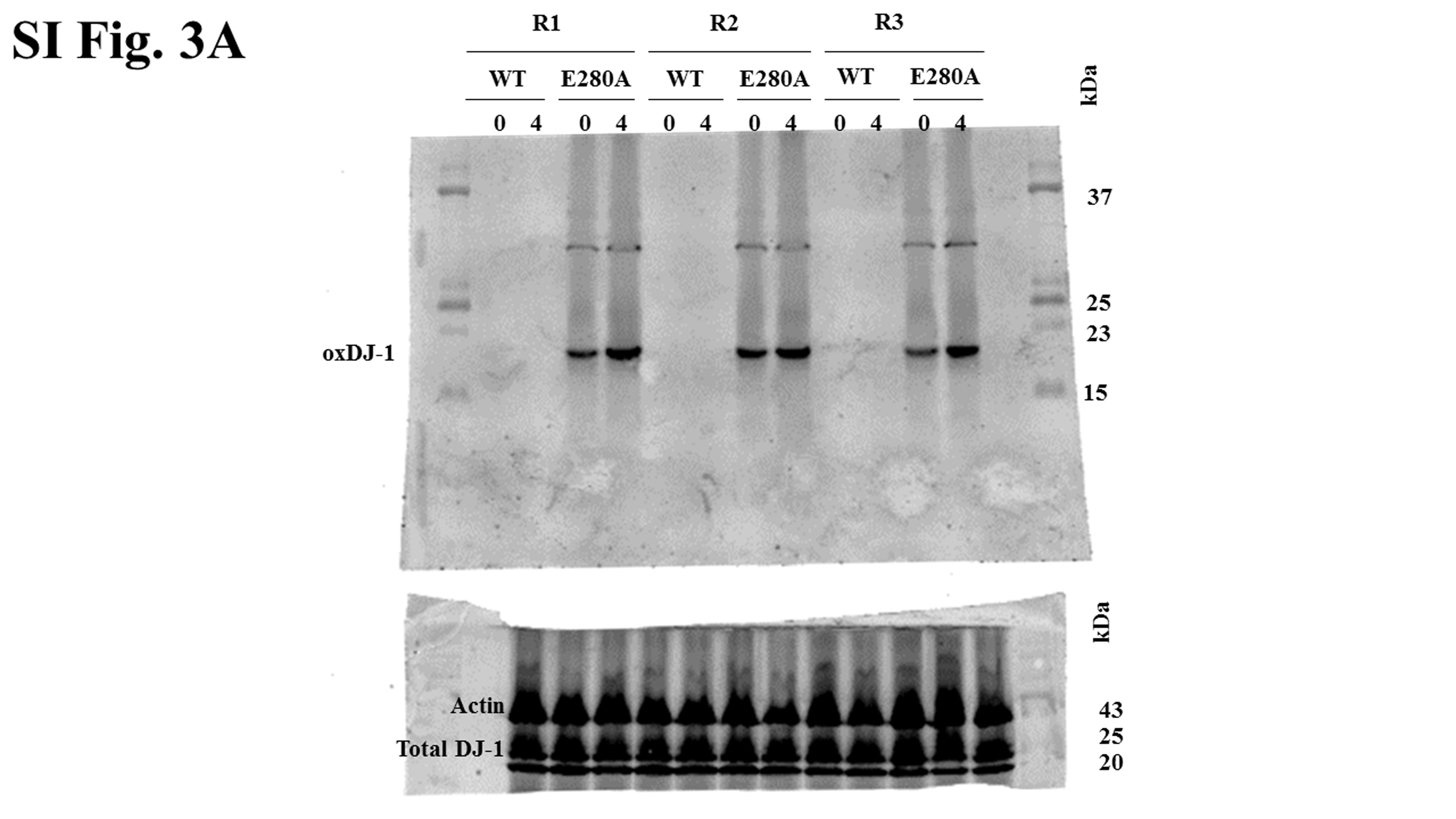

Supplement: S1 File — (ZIP) [file pone.0221669.s006.zip › 300dpi Support Info/S5 I.tif]

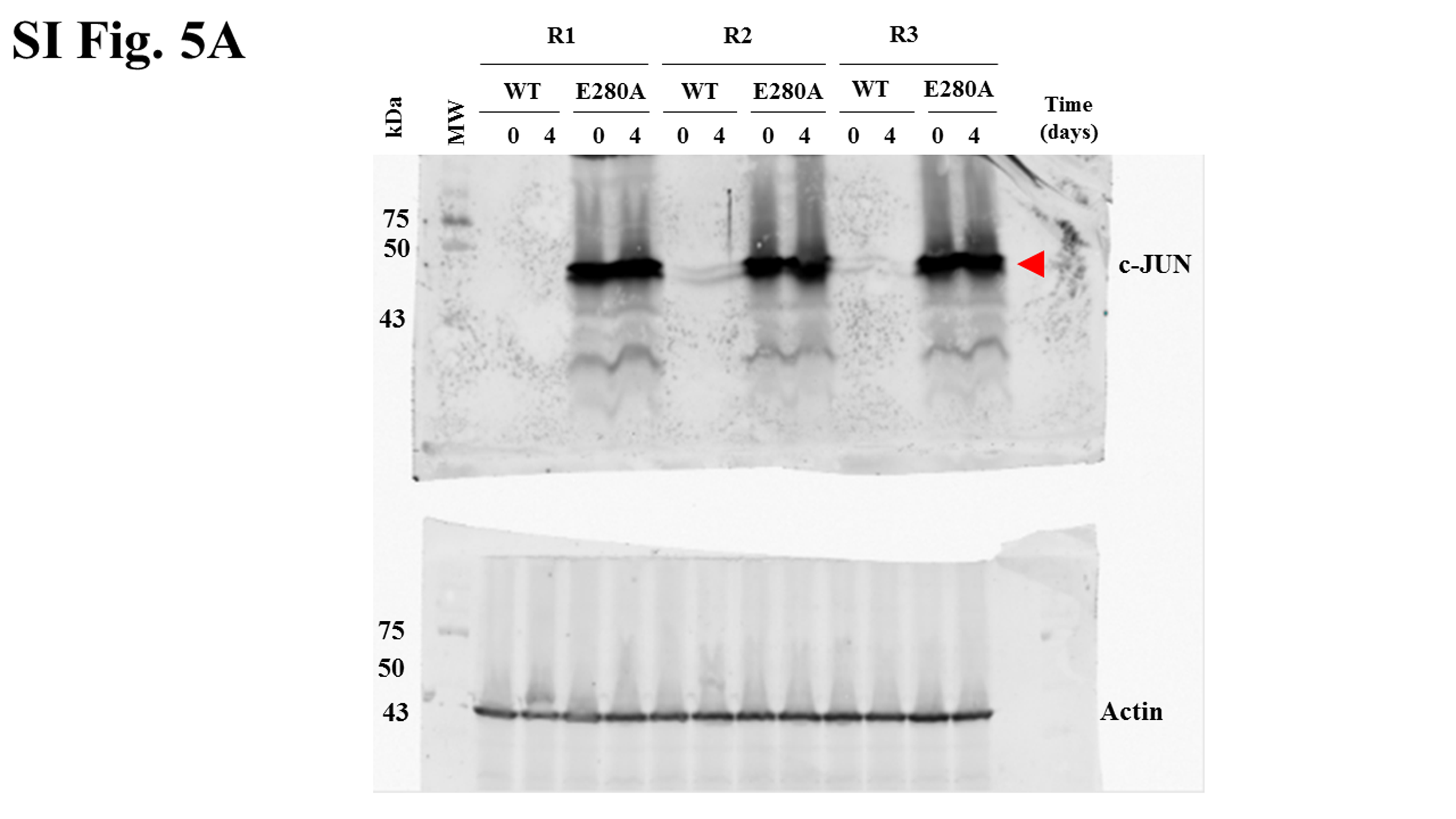

Supplement: S1 File — (ZIP) [file pone.0221669.s006.zip › 300dpi Support Info/S6 I.tif]

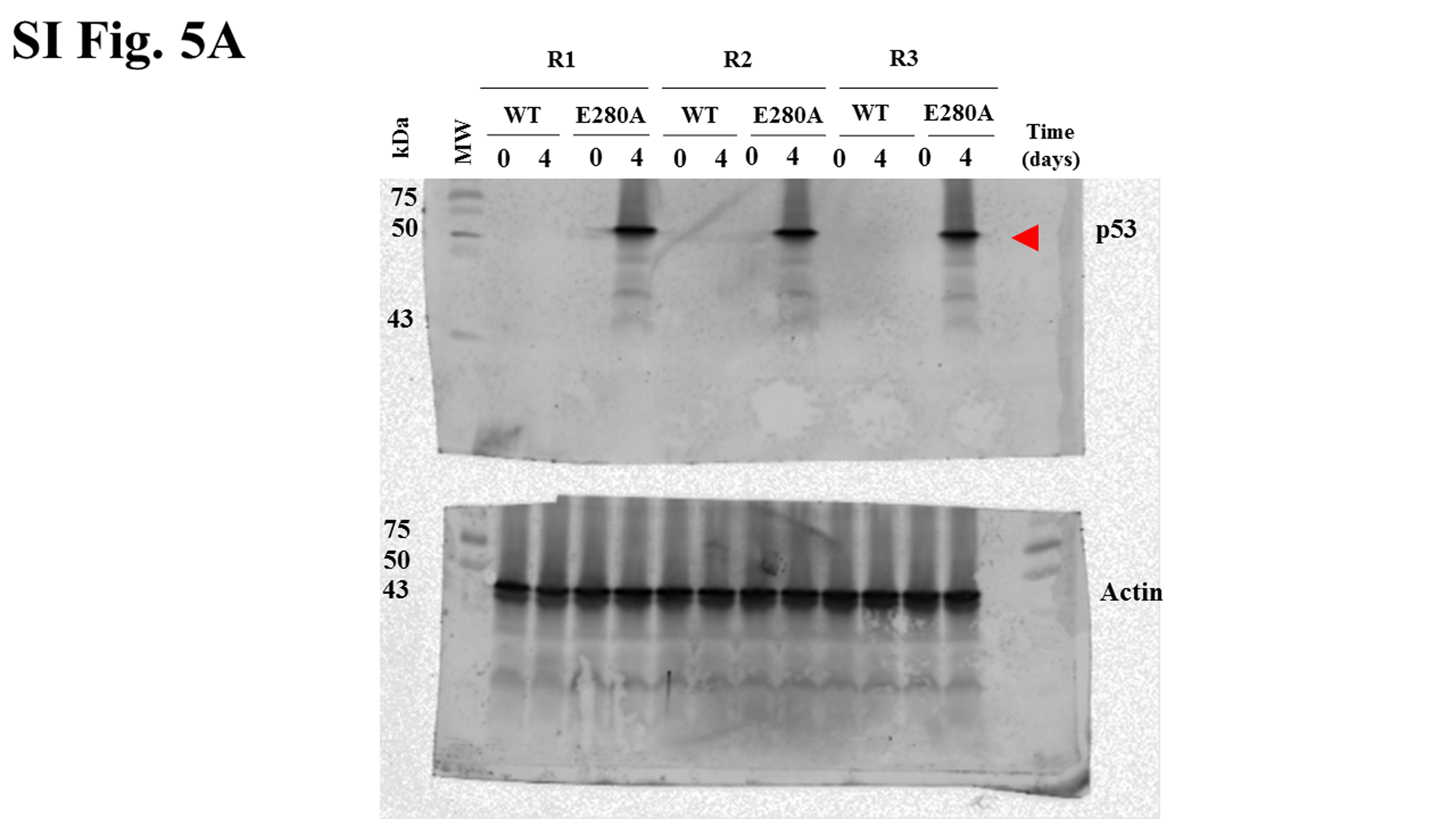

Supplement: S1 File — (ZIP) [file pone.0221669.s006.zip › 300dpi Support Info/S7 I.tif]

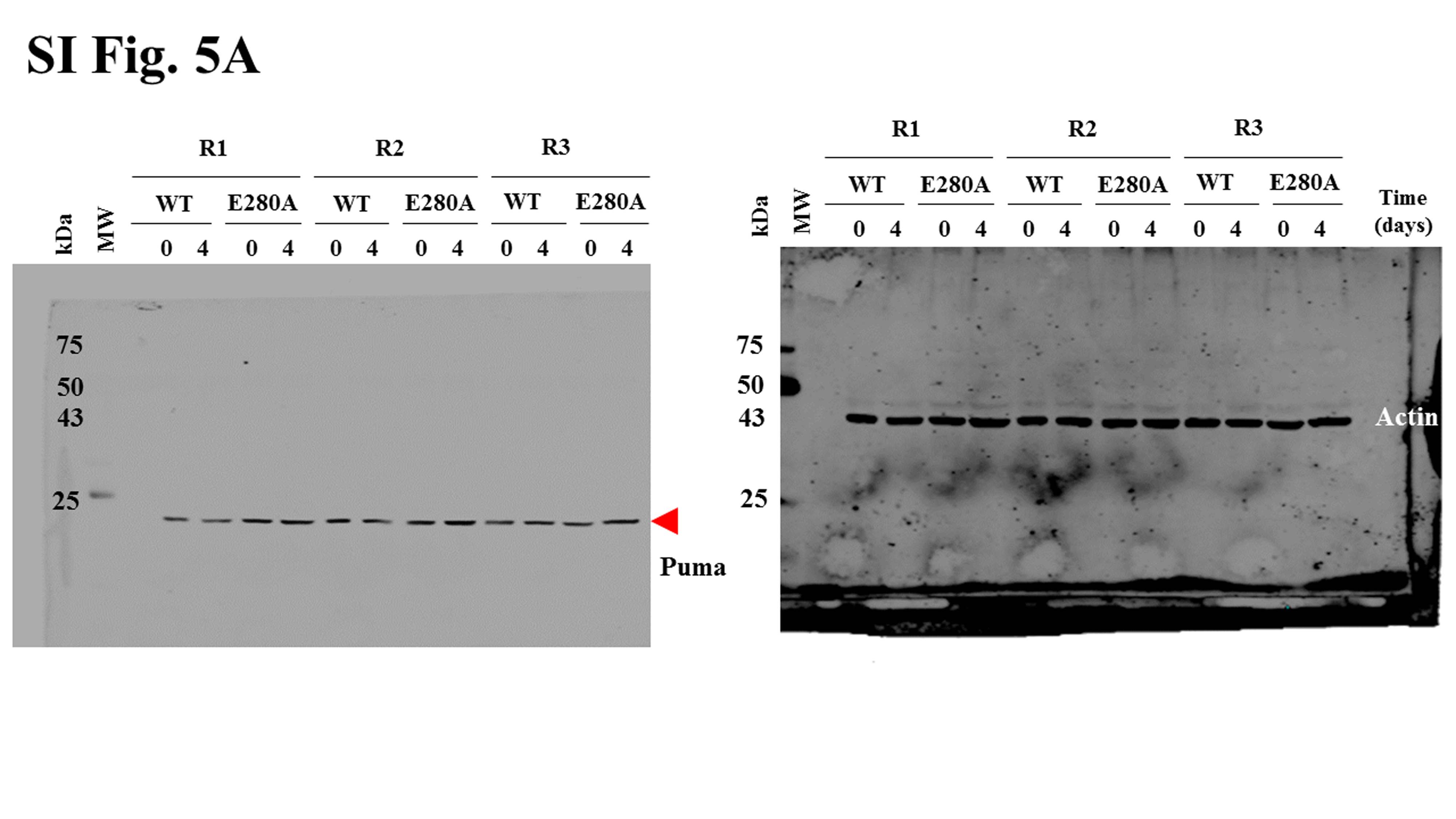

Supplement: S1 File — (ZIP) [file pone.0221669.s006.zip › 300dpi Support Info/S8 I.tif]

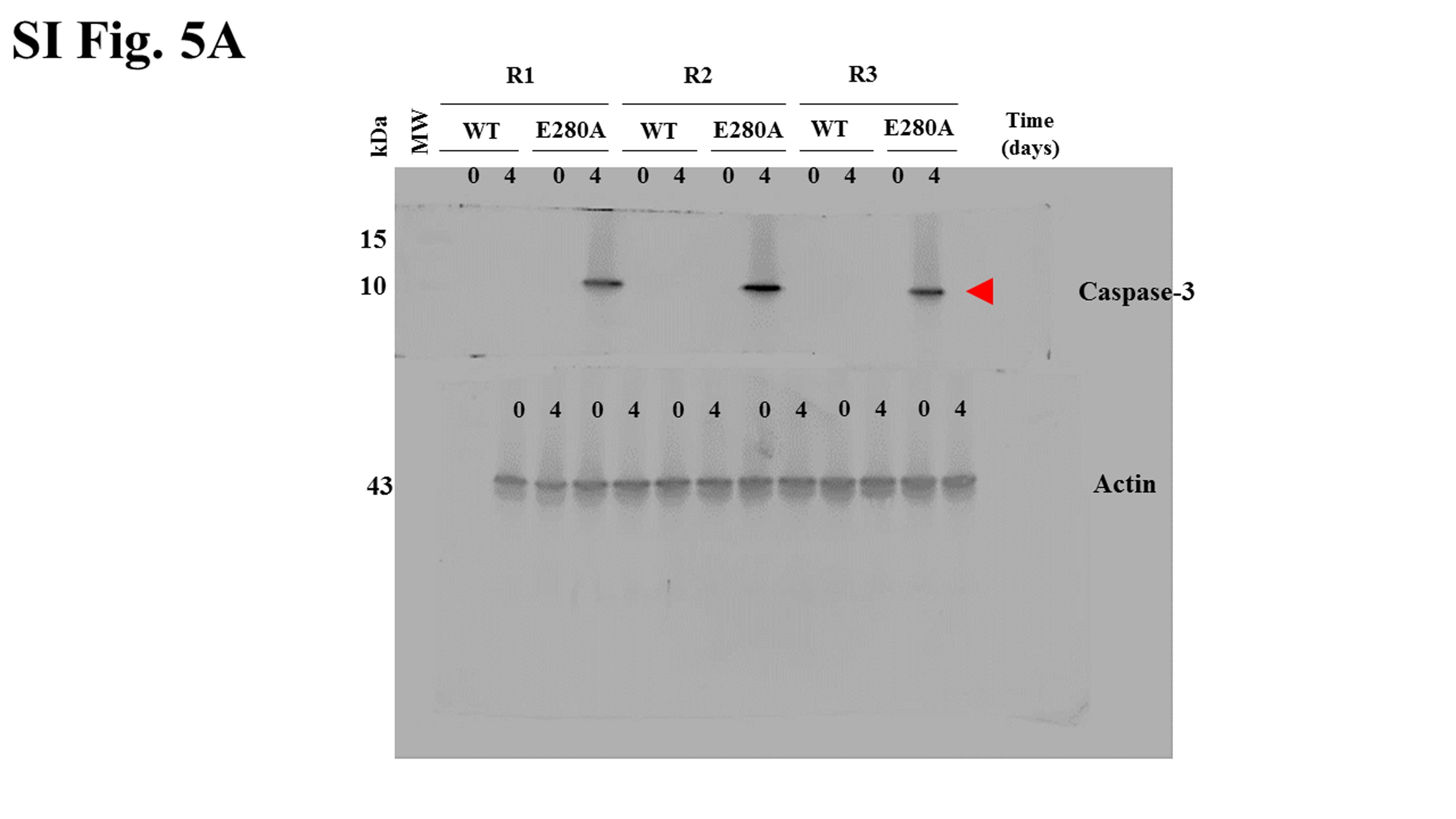

Supplement: S1 File — (ZIP) [file pone.0221669.s006.zip › 300dpi Support Info/S9 I.tif]
