# Supplementary material for: Cholinergic-like neurons carrying PSEN1 E280A mutation from familial Alzheimer’s disease reveal intraneuronal sAPPβ fragments accumulation, hyperphosphorylation of TAU, oxidative stress, apoptosis and Ca2+ dysregulation: Therapeutic implications
Source: PLoS One. 2020 May 21;15(5):e0221669. doi: 10.1371/journal.pone.0221669 (PMC7241743; doi:10.1371/journal.pone.0221669)
Supplement: S5 Fig — Mascot Search Results of (A) MS/MS fragmentation of LPTTAASTPDAVDK, and (B) MS/MS fragmentation of AVIQHFQEK. (C) List of proteins identified in the PAGE gel region of high molecular weight between 60 and 125 kDa. (PDF) [file pone.0221669.s005.pdf]

(a)

Mascot Search Results

Peptide View

MS/MS Fragmentation of **LPTTAASTPDAVDK**
Found in **sp|P05067|A4_HUMAN**, sp|P05067|A4_HUMAN Amyloid-beta precursor protein OS=Homo sapiens OX=9606 GN=APP PE=1 SV=3

Match to Query 11590: 1385.704544 from(693.859548,2+)
Title: carlosdda_2.04888.04888.2.dta
Data file D:\proj\20200124-104754-carlos\carlosdda_2.mgf

Click mouse within plot area to zoom in by factor of two about that point
Or, Plot from 100 to 1300 Da Full range

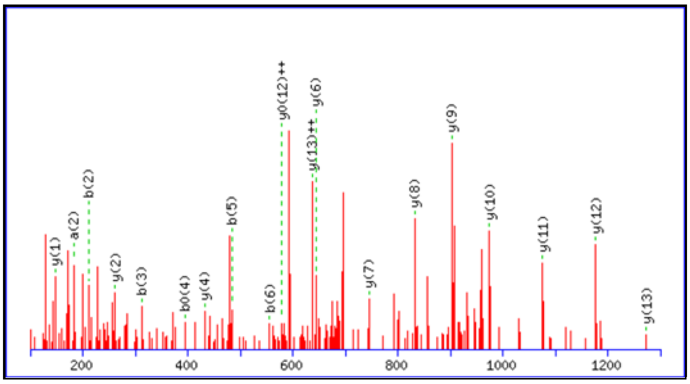

Monoisotopic mass of neutral peptide Mr(calc): 1385.7038
Ions Score: 52 **Expect:** 0.00084
Matches (Bold Red): 19/186 fragment ions using 60 most intense peaks

#	Immon.	a	a++	a0	a0++	b	b++	b0	b0++	Seq.	y	y++	y*	y*++	y0	y0++	#
1	86.0964	86.0964	43.5519			114.0913	57.5493			L							14
2	70.0651	**183.1492**	92.0782			**211.1441**	106.0757			P	**1273.6270**	**637.3172**	1256.6005	628.8039	1255.6165	628.3119	13
3	74.0600	284.1969	142.6021	266.1863	133.5968	**312.1918**	156.5995	294.1812	147.5942	T	**1176.5743**	588.7908	1159.5477	580.2775	1158.5637	**579.7855**	12
4	74.0600	385.2445	193.1259	367.2340	184.1206	413.2395	207.1234	**395.2289**	198.1181	T	**1075.5266**	538.2669	1058.5000	529.7537	1057.5160	529.2617	11
5	44.0495	456.2817	228.6445	438.2711	219.6392	**484.2766**	242.6419	466.2660	233.6366	A	**974.4789**	487.7431	957.4524	479.2298	956.4684	478.7378	10
6	44.0495	527.3188	264.1630	509.3082	255.1577	**555.3137**	278.1605	537.3031	269.1552	A	**903.4418**	452.2245	886.4153	443.7113	885.4312	443.2193	9
7	60.0444	614.3508	307.6790	596.3402	298.6738	642.3457	321.6765	624.3352	312.6712	S	**832.4047**	416.7060	815.3781	408.1927	814.3941	407.7007	8
8	74.0600	715.3985	358.2029	697.3879	349.1976	743.3934	372.2003	725.3828	363.1951	T	**745.3727**	373.1900	728.3461	364.6767	727.3621	364.1847	7
9	70.0651	812.4512	406.7293	794.4407	397.7240	840.4462	420.7267	822.4356	411.7214	P	**644.3250**	322.6661	627.2984	314.1529	626.3144	313.6608	6
10	88.0393	927.4782	464.2427	909.4676	455.2375	955.4731	478.2402	937.4625	469.2349	D	547.2722	274.1397	530.2457	265.6265	529.2617	265.1345	5
11	44.0495	998.5153	499.7613	980.5047	490.7560	1026.5102	513.7587	1008.4997	504.7535	A	**432.2453**	216.6263	415.2187	208.1130	414.2347	207.6210	4
12	72.0808	1097.5837	549.2955	1079.5732	540.2902	1125.5786	563.2930	1107.5681	554.2877	V	361.2082	181.1077	344.1816	172.5944	343.1976	172.1024	3
13	88.0393	1212.6107	606.8090	1194.6001	597.8037	1240.6056	620.8064	1222.5950	611.8011	D	**262.1397**	131.5735	245.1132	123.0602	244.1292	122.5682	2
14	101.1073									K	**147.1128**	74.0600	130.0863	65.5468			1

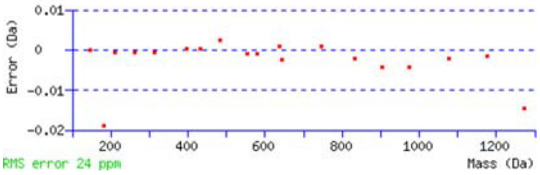

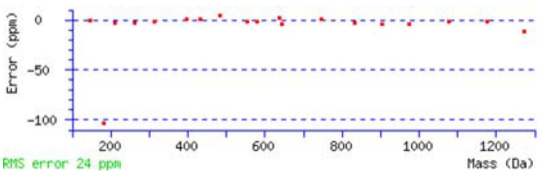

NCBI **BLAST** search of LPTTAASTPDAVDK
(Parameters: blastp, nr protein database, expect=20000, no filter, PAM30)
Other BLAST web gateways

All matches to this query

Score	Mr(calc):	Delta	Sequence
52.1	1385.7038	0.0007	LPTTAASTPDAVDK

Mascot: http://www.matrixscience.com/

(b)

Mascot Search Results

Peptide View

MS/MS Fragmentation of **AVIQHFQEK**
Found in **sp|P05067|A4_HUMAN**, sp|P05067|A4_HUMAN Amyloid-beta precursor protein OS=Homo sapiens OX=9606 GN=APP PE=1 SV=3

Match to Query 4829: 1098.584769 from(367.202199,3+)
Title: carlosdda_2.02864.02864.3.dta
Data file D:\proj\20200124-104754-carlos\carlosdda_2.mgf

Click mouse within plot area to zoom in by factor of two about that point
Or, Plot from 50 to 850 Da Full range

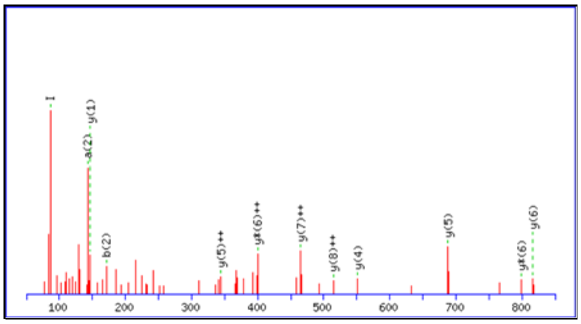

Monoisotopic mass of neutral peptide Mr(calc): 1098.5822
Ions Score: 23 **Expect:** 0.48
Matches (Bold Red): 12/111 fragment ions using 21 most intense peaks

#	Immon.	a	a++	a*	a*++	a0	a0++	b	b++	b*	b*++	b0	b0++	Seq.	y	y++	y*	y*++	y0	y0++	#
1	44.0495	44.0495	22.5284					72.0444	36.5258					A							9
2	72.0808	**143.1179**	72.0626					**171.1128**	86.0600					V	1028.5524	**514.7798**	1011.5258	506.2665	1010.5418	505.7745	8
3	**86.0964**	256.2020	128.6046					284.1969	142.6021					I	929.4839	**465.2456**	912.4574	456.7323	911.4734	456.2403	7
4	101.0709	384.2605	192.6339	367.2340	184.1206			412.2554	206.6314	395.2289	198.1181			Q	**816.3999**	408.7036	**799.3733**	**400.1903**	798.3893	399.6983	6
5	110.0713	521.3194	261.1634	504.2929	252.6501			549.3144	275.1608	532.2878	266.6475			H	**688.3413**	**344.6743**	671.3148	336.1610	670.3307	335.6690	5
6	120.0808	668.3879	334.6976	651.3613	326.1843			696.3828	348.6950	679.3562	340.1817			F	**551.2824**	276.1448	534.2558	267.6316	533.2718	267.1395	4
7	101.0709	796.4464	398.7269	779.4199	390.2136			824.4414	412.7243	807.4148	404.2110			Q	404.2140	202.6106	387.1874	194.0974	386.2034	193.6053	3
8	102.0550	925.4890	463.2482	908.4625	454.7349	907.4785	454.2429	953.4839	477.2456	936.4574	468.7323	935.4734	468.2403	E	276.1554	138.5813	259.1288	130.0681	258.1448	129.5761	2
9	101.1073													K	**147.1128**	74.0600	130.0863	65.5468			1

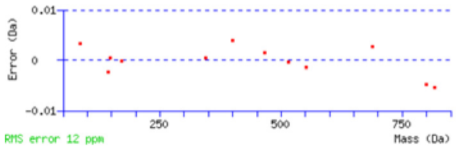

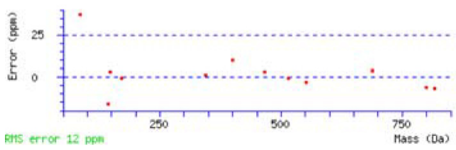

NCBI **BLAST** search of AVIQHFQEK
(Parameters: blastp, nr protein database, expect=20000, no filter, PAM30)
Other BLAST web gateways

All matches to this query

Score	Mr(calc):	Delta	Sequence
22.7	1098.5822	0.0026	AVIQHFQEK
0.4	1098.5855	-0.0008	KQLPAKCGVN
0.1	1098.5855	-0.0008	QPPKRMLTS

Mascot: http://www.matrixscience.com/

(c)

Experiment: 20200128-carlosdda_2-human-unspecific 50% probability, 1 peptide minimum, displaying Percent Coverage

Probability Legend:
- over 95%
- 80% to 94%
- 50% to 79%
- 20% to 49%
- 0% to 19%

#	Visible?	Starred?	BioView: Identified Proteins (152)
1	☑	☆	Isoform 3 of Alpha-actinin-1 OS=Homo sapiens OX=9606 GN=ACTN1
2	☑	☆	Endoplasmin OS=Homo sapiens OX=9606 GN=HSP90B1 PE=1 SV=1
3	☑	☆	Isoform 2 of Filamin-A OS=Homo sapiens OX=9606 GN=FLNA
4	☑	☆	Alpha-actinin-4 OS=Homo sapiens OX=9606 GN=ACTN4 PE=1 SV=2
5	☑	☆	Serum albumin OS=Bos taurus GN=ALB PE=1 SV=4
6	☑	☆	Elongation factor 2 OS=Homo sapiens OX=9606 GN=EEF2 PE=1 SV=4
7	☑	☆	retention time standard
8	☑	☆	Transitional endoplasmic reticulum ATPase OS=Homo sapiens OX=9606 GN=VCP PE=1 SV=4
9	☑	☆	Isoform 2 of Procollagen-lysine,2-oxoglutarate 5-dioxygenase 2 OS=Homo sapiens OX=9606 GN=PLOD2
10	☑	☆	Isoform 2 of Filamin-B OS=Homo sapiens OX=9606 GN=FLNB
11	☑	☆	Isoform 2 of Heat shock protein HSP 90-alpha OS=Homo sapiens OX=9606 GN=HSP90AA1
12	☑	☆	Isoform 1 of Vinculin OS=Homo sapiens OX=9606 GN=VCL
13	☑	☆	Isoform 2 of Ubiquitin-like modifier-activating enzyme 1 OS=Homo sapiens OX=9606 GN=UBA1
14	☑	☆	Ribosome-binding protein 1 OS=Homo sapiens OX=9606 GN=RRBP1 PE=1 SV=5
15	☑	☆	Integrin beta-1 OS=Homo sapiens OX=9606 GN=ITGB1 PE=1 SV=2
16	☑	☆	Importin subunit beta-1 OS=Homo sapiens OX=9606 GN=KPNB1 PE=1 SV=2
17	☑	☆	IQ motif containing GTPase activating protein 1 OS=Homo sapiens OX=9606 GN=IQGAP1 PE=1 SV=1
18	☑	☆	Isoform 2 of Neutral alpha-glucosidase AB OS=Homo sapiens OX=9606 GN=GANAB
19	☑	☆	Isoform 3 of Importin-5 OS=Homo sapiens OX=9606 GN=IPO5
20	☑	☆	Alanine--tRNA ligase, cytoplasmic OS=Homo sapiens OX=9606 GN=AARS PE=1 SV=2
21	☑	☆	Myosin-9 OS=Homo sapiens OX=9606 GN=MYH9 PE=1 SV=4
22	☑	☆	Mitochondrial 10-formyltetrahydrofolate dehydrogenase OS=Homo sapiens OX=9606 GN=ALDH1L2 PE=1 SV=2
23	☑	☆	
24	☑	☆	Isoform Beta of Signal transducer and activator of transcription 1-alpha/beta OS=Homo sapiens OX=9606 GN=STAT1
25	☑	☆	Isoform 2 of 4F2 cell-surface antigen heavy chain OS=Homo sapiens OX=9606 GN=SLC3A2
26	☑	☆	Glycogen phosphorylase, brain form OS=Homo sapiens OX=9606 GN=PYGB PE=1 SV=5
27	☑	☆	Cytoplasmic aconitate hydratase OS=Homo sapiens OX=9606 GN=ACO1 PE=1 SV=3
28	☑	☆	Puromycin-sensitive aminopeptidase OS=Homo sapiens OX=9606 GN=NPEPPS PE=1 SV=2
29	☑	☆	Major vault protein OS=Homo sapiens OX=9606 GN=MVP PE=1 SV=4
30	☑	☆	Isoform 3 of Exportin-2 OS=Homo sapiens OX=9606 GN=CSE1L
31	☑	☆	Hexokinase-1 OS=Homo sapiens OX=9606 GN=HK1 PE=1 SV=3
32	☑	☆	cDNA FLJ59430, highly similar to Protein disulfide-isomerase OS=Homo sapiens OX=9606 PE=2 SV=1
33	☑	☆	Coatomer subunit gamma-1 OS=Homo sapiens OX=9606 GN=COPG1 PE=1 SV=1
34	☑	☆	Cullin-associated NEDD8-dissociated protein 1 OS=Homo sapiens OX=9606 GN=CAND1 PE=1 SV=2
35	☑	☆	Staphylococcal nuclease domain-containing protein 1 OS=Homo sapiens OX=9606 GN=SND1 PE=1 SV=1
36	☑	☆	Isoform 2 of AP-2 complex subunit beta OS=Homo sapiens OX=9606 GN=AP2B1
37	☑	☆	Catenin alpha-1 OS=Homo sapiens OX=9606 GN=CTNNA1 PE=1 SV=1
38	☑	☆	Heat shock protein HSP 90-beta OS=Homo sapiens OX=9606 GN=HSP90AB1 PE=1 SV=4
39	☑	☆	Neprilysin OS=Homo sapiens OX=9606 GN=MME PE=1 SV=2
40	☑	☆	Isoleucine--tRNA ligase, mitochondrial OS=Homo sapiens OX=9606 GN=IARS2 PE=1 SV=2
41	☑	☆	Keratin, type II cytoskeletal 1 OS=Homo sapiens OX=9606 GN=KRT1 PE=1 SV=6
42	☑	☆	Isoform 2 of Programmed cell death 6-interacting protein OS=Homo sapiens OX=9606 GN=PDCD6IP
43	☑	☆	Isoform 2 of Calnexin OS=Homo sapiens OX=9606 GN=CANX
44	☑	☆	Heat shock 70 kDa protein 4 OS=Homo sapiens OX=9606 GN=HSPA4 PE=1 SV=4
45	☑	☆	Isoform 2 of Protein transport protein Sec24D OS=Homo sapiens OX=9606 GN=SEC24D
46	☑	☆	Protein-glutamine gamma-glutamyltransferase 2 OS=Homo sapiens OX=9606 GN=TGM2 PE=1 SV=2
47	☑	☆	Isoform M1 of Pyruvate kinase PKM OS=Homo sapiens OX=9606 GN=PKM
48	☑	☆	Isoform 2 of Lon protease homolog, mitochondrial OS=Homo sapiens OX=9606 GN=LONP1
49	☑	☆	Protein disulfide-isomerase A4 OS=Homo sapiens OX=9606 GN=PDIA4 PE=1 SV=2
50	☑	☆	Talin-1 OS=Homo sapiens OX=9606 GN=TLN1 PE=1 SV=3
51	☑	☆	Isoform 2 of Transportin-1 OS=Homo sapiens OX=9606 GN=TNPO1
52	☑	☆	Isoform 2 of Integrin alpha-V OS=Homo sapiens OX=9606 GN=ITGAV
53	☑	☆	Elongation factor 1-alpha 1 OS=Homo sapiens OX=9606 GN=EEF1A1 PE=1 SV=1
54	☑	☆	Isoform 2 of General vesicular transport factor p115 OS=Homo sapiens OX=9606 GN=USO1
55	☑	☆	Isoform 2 of Glycogen phosphorylase, liver form OS=Homo sapiens OX=9606 GN=PYGL
56	☑	☆	Cytoskeleton-associated protein 4 OS=Homo sapiens OX=9606 GN=CKAP4 PE=1 SV=2
57	☑	☆	Glycine--tRNA ligase OS=Homo sapiens OX=9606 GN=GARS PE=1 SV=3
58	☑	☆	Vacuolar protein sorting-associated protein 35 OS=Homo sapiens OX=9606 GN=VPS35 PE=1 SV=2
59	☑	☆	Isoform TGN46 of Trans-Golgi network integral membrane protein 2 OS=Homo sapiens OX=9606 GN=TGOLN2
60	☑	☆	C-1-tetrahydrofolate synthase, cytoplasmic OS=Homo sapiens OX=9606 GN=MTHFD1 PE=1 SV=3

	Accession Number	Molecular Weight	Protein Grouping Ambiguity	carlosdda_2
	sp|P12814-3|ACTN1_HUMAN (+2)	106 kDa	★	27%
	sp|P14625|ENPL_HUMAN (+2)	92 kDa	★	26%
	sp|P21333-2|FLNA_HUMAN (+3)	280 kDa	★	15%
	sp|O43707|ACTN4_HUMAN (+1)	105 kDa	★	25%
	sp|P02769|ALBU_BOVIN	69 kDa		26%
	sp|P13639|EF2_HUMAN (+1)	95 kDa		11%
	ST|RTSTANDARD	19 kDa		74%
	sp|P55072|TERA_HUMAN (+1)	89 kDa		13%
	sp|O00469-2|PLOD2_HUMAN	87 kDa		12%
	sp|O75369-2|FLNB_HUMAN (+3)	276 kDa	★	7.5%
	sp|P07900-2|HS90A_HUMAN (+2)	98 kDa	★	6.8%
	sp|P18206-2|VINC_HUMAN (+4)	117 kDa		11%
	sp|P22314-2|UBA1_HUMAN (+2)	114 kDa		9.4%
	sp|Q9P2E9|RRBP1_HUMAN (+3)	152 kDa		12%
	sp|P05556|ITB1_HUMAN	88 kDa		15%
	sp|Q14974|IMB1_HUMAN (+1)	97 kDa		11%
	tr|A4QPB0|A4QPB0_HUMAN	189 kDa		6.5%
	sp|Q14697-2|GANAB_HUMAN	109 kDa		4.3%
	sp|O00410-3|IPO5_HUMAN (+3)	126 kDa		7.9%
	sp|P49588|SYAC_HUMAN	107 kDa		13%
	sp|P35579|MYH9_HUMAN (+2)	227 kDa		5.5%
	sp|Q3SY69|AL1L2_HUMAN (+1)	102 kDa		9.5%
	sp|P11021|BIP_HUMAN (+1)	72 kDa		12%
	sp|P42224-2|STAT1_HUMAN (+2)	83 kDa		6.9%
	sp|P08195-2|4F2_HUMAN (+5)	58 kDa		17%
	sp|P11216|PYGB_HUMAN (+1)	97 kDa		6.3%
	sp|P21399|ACOC_HUMAN (+1)	98 kDa		5.2%
	sp|P55786|PSA_HUMAN (+1)	103 kDa		5.0%
	sp|Q14764|MVP_HUMAN (+2)	99 kDa		8.3%
	sp|P55060-3|XPO2_HUMAN (+2)	108 kDa		6.9%
	sp|P19367|HXK1_HUMAN (+3)	102 kDa	★	2.4%
	tr|B4DUA5|B4DUA5_HUMAN (+1)	51 kDa		11%
	sp|Q9Y678|COPG1_HUMAN	98 kDa		7.8%
	sp|Q86VP6|CAND1_HUMAN (+1)	136 kDa		4.1%
	sp|Q7KZF4|SND1_HUMAN (+2)	102 kDa		2.9%
	sp|P63010-2|AP2B1_HUMAN (+5)	106 kDa	★	1.2%
	sp|P35221|CTNA1_HUMAN (+2)	100 kDa		10%
	sp|P08238|HS90B_HUMAN (+2)	83 kDa	★	7.9%
	sp|P08473|NEP_HUMAN (+1)	86 kDa		6.0%
	sp|Q9NSE4|SYIM_HUMAN (+1)	114 kDa		5.7%
	sp|P04264|K2C1_HUMAN (+6)	66 kDa	★	5.1%
	sp|Q8WUM4-2|PDC6I_HUMAN (+2)	97 kDa		3.7%
	sp|P27824-2|CALX_HUMAN (+2)	72 kDa		6.2%
	sp|P34932|HSP74_HUMAN (+2)	94 kDa		6.4%
	sp|O94855-2|SC24D_HUMAN (+2)	113 kDa		2.7%
	sp|P21980|TGM2_HUMAN (+3)	77 kDa		1.9%
	sp|P14618-2|KPYM_HUMAN (+4)	58 kDa		12%
	sp|P36776-2|LONM_HUMAN (+7)	100 kDa		2.7%
	sp|P13667|PDIA4_HUMAN (+1)	73 kDa		7.8%
	sp|Q9Y490|TLN1_HUMAN (+1)	270 kDa		1.9%
	sp|Q92973-2|TNPO1_HUMAN (+3)	101 kDa		2.5%
	sp|P06756-2|ITAV_HUMAN (+4)	112 kDa		2.7%
	sp|P68104|EF1A1_HUMAN (+14)	50 kDa		2.4%
	sp|O60763-2|USO1_HUMAN (+2)	109 kDa		4.8%
	sp|P06737-2|PYGL_HUMAN (+2)	93 kDa		7.0%
	sp|Q07065|CKAP4_HUMAN (+4)	66 kDa		2.0%
	sp|P41250|GARS_HUMAN (+1)	83 kDa		4.1%
	sp|Q96QK1|VPS35_HUMAN (+1)	92 kDa		3.8%
	sp|O43493-2|TGON2_HUMAN (+4)	46 kDa		6.9%
	sp|P11586|C1TC_HUMAN (+4)	102 kDa		1.2%

Probability Legend:

- over 95%
- 80% to 94%
- 50% to 79%
- 20% to 49%
- 0% to 19%

#	Visible?	Starred?	Bio View: Identified Proteins (152)
61	☑	☆	Collagen alpha-1(III) chain OS=Homo sapiens OX=9606 GN=COL3A1 PE=1 SV=4
62	☑	☆	Heat shock cognate 71 kDa protein OS=Homo sapiens OX=9606 GN=HSPA8 PE=1 SV=1
63	☑	☆	cDNA FLJ59408, highly similar to 150 kDa oxygen-regulated protein (Orp150) OS=Homo sapiens OX=9606 PE=2 SV=1
64	☑	☆	2-oxoglutarate dehydrogenase, mitochondrial OS=Homo sapiens OX=9606 GN=OGDH PE=1 SV=3
65	☑	☆	cDNA FLJ13414 fis, clone PLACE1001748, highly similar to Homo sapiens pitrilysin metallopeptidase 1 (PITRM1), mRNA OS=Homo sapiens OX=9606 PE=
66	☑	☆	Isoform 2 of Filamin-C OS=Homo sapiens OX=9606 GN=FLNC
67	☑	☆	Collagen alpha-1(VI) chain OS=Homo sapiens OX=9606 GN=COL6A1 PE=1 SV=3
68	☑	☆	Hexokinase-2 OS=Homo sapiens OX=9606 GN=HK2 PE=1 SV=2
69	☑	☆	Isoform Short of Ubiquitin carboxyl-terminal hydrolase 5 OS=Homo sapiens OX=9606 GN=USP5
70	☑	☆	Threonine--tRNA ligase, cytoplasmic OS=Homo sapiens OX=9606 GN=TARS PE=1 SV=3
71	☑	☆	26S proteasome non-ATPase regulatory subunit 2 OS=Homo sapiens OX=9606 GN=PSMD2 PE=1 SV=3
72	☑	☆	Keratin, type I cytoskeletal 10 OS=Homo sapiens OX=9606 GN=KRT10 PE=1 SV=6
73	☑	☆	Microtubule-associated protein 1B OS=Homo sapiens OX=9606 GN=MAP1B PE=1 SV=2
74	☑	☆	Caldesmon OS=Homo sapiens OX=9606 GN=CALD1 PE=1 SV=1
75	☑	☆	ATP-dependent RNA helicase DDX1 OS=Homo sapiens OX=9606 GN=DDX1 PE=1 SV=2
76	☑	☆	Isoform 10 of Aspartyl/asparaginyl beta-hydroxylase OS=Homo sapiens OX=9606 GN=ASPH
77	☑	☆	Insulin-degrading enzyme OS=Homo sapiens OX=9606 GN=IDE PE=1 SV=4
78	☑	☆	Actin, cytoplasmic 1 OS=Homo sapiens OX=9606 GN=ACTB PE=1 SV=1
79	☑	☆	Annexin A1 OS=Homo sapiens OX=9606 GN=ANXA1 PE=1 SV=2
80	☑	☆	Vigilin OS=Homo sapiens OX=9606 GN=HDLBP PE=1 SV=2
81	☑	☆	Isoform 2 of Myoferlin OS=Homo sapiens OX=9606 GN=MYOF
82	☑	☆	Lysosome membrane protein 2 OS=Homo sapiens OX=9606 GN=SCARB2 PE=1 SV=2
83	☑	☆	Isoform 3 of MICOS complex subunit MIC60 OS=Homo sapiens OX=9606 GN=IMMT
84	☑	☆	Alpha-aminoadipic semialdehyde synthase, mitochondrial OS=Homo sapiens OX=9606 GN=AASS PE=1 SV=1
85	☑	☆	Alpha-enolase OS=Homo sapiens OX=9606 GN=ENO1 PE=1 SV=1
86	☑	☆	Moesin OS=Homo sapiens OX=9606 GN=MSN PE=1 SV=3
87	☑	☆	Aconitate hydratase, mitochondrial OS=Homo sapiens OX=9606 GN=ACO2 PE=1 SV=2
88	☑	☆	cDNA FLJ55432, highly similar to Probable ATP-dependent RNA helicase DDX58 OS=Homo sapiens OX=9606 PE=2 SV=1
89	☑	☆	Transgelin OS=Homo sapiens OX=9606 GN=TAGLN PE=1 SV=4
90	☑	☆	Kinesin-1 heavy chain OS=Homo sapiens OX=9606 GN=KIF5B PE=1 SV=1
91	☑	☆	Trifunctional enzyme subunit alpha, mitochondrial OS=Homo sapiens OX=9606 GN=HADHA PE=1 SV=2
92	☑	☆	Isoform LCRMP-1 of Dihydropyrimidinase-related protein 1 OS=Homo sapiens OX=9606 GN=CRMP1
93	☑	☆	Keratin, type II cytoskeletal 2 epidermal OS=Homo sapiens OX=9606 GN=KRT2 PE=1 SV=2
94	☑	☆	Isoform 2 of Collagen alpha-3(VI) chain OS=Homo sapiens OX=9606 GN=COL6A3
95	☑	☆	Isoform 2 of CD166 antigen OS=Homo sapiens OX=9606 GN=ALCAM
96	☑	☆	Isoform Beta-3B of Integrin beta-3 OS=Homo sapiens OX=9606 GN=ITGB3
97	☑	☆	Dihydropyrimidine dehydrogenase [NADP(+)] OS=Homo sapiens OX=9606 GN=DPYD PE=1 SV=2
98	☑	☆	Keratin 14 (Epidermolysis bullosa simplex, Dowling-Meara, Koebner), isoform CRA_a OS=Homo sapiens OX=9606 GN=KRT14 PE=3 SV=1
99	☑	☆	Isoform 2 of UDP-glucose:glycoprotein glucosyltransferase 1 OS=Homo sapiens OX=9606 GN=UGGT1
100	☑	☆	Laminin subunit gamma-1 OS=Homo sapiens OX=9606 GN=LAMC1 PE=1 SV=3
101	☑	☆	Monofunctional C1-tetrahydrofolate synthase, mitochondrial OS=Homo sapiens OX=9606 GN=MTHFD1L PE=1 SV=1
102	☑	☆	cDNA FLJ56935, highly similar to Centromere/kinetochore protein zw10 homolog OS=Homo sapiens OX=9606 PE=2 SV=1
103	☑	☆	Exportin-1 OS=Homo sapiens OX=9606 GN=XPO1 PE=1 SV=1
104	☑	☆	Isoform 2 of ELKS/Rab6-interacting/CAST family member 1 OS=Homo sapiens OX=9606 GN=ERC1
105	☑	☆	Isoform Beta of Heat shock protein 105 kDa OS=Homo sapiens OX=9606 GN=HSPH1
106	☑	☆	Intercellular adhesion molecule 1 OS=Homo sapiens OX=9606 GN=ICAM1 PE=1 SV=2
107	☑	☆	Isoform 2 of Tubulin alpha-3C chain OS=Homo sapiens OX=9606 GN=TUBA3C
108	☑	☆	Methionine--tRNA ligase, cytoplasmic OS=Homo sapiens OX=9606 GN=MARS PE=1 SV=2
109	☑	☆	Isoform 2 of Calcium-transporting ATPase type 2C member 1 OS=Homo sapiens OX=9606 GN=ATP2C1
110	☑	☆	Nucleolin OS=Homo sapiens OX=9606 GN=NCL PE=1 SV=3
111	☑	☆	Leucine zipper-EF-hand containing transmembrane protein 1, isoform CRA_a OS=Homo sapiens OX=9606 GN=LETM1 PE=4 SV=1
112	☑	☆	Isoform 4 of Dynamin-like 120 kDa protein, mitochondrial OS=Homo sapiens OX=9606 GN=OPA1
113	☑	☆	Isoform 2 of 1-phosphatidylinositol 4,5-bisphosphate phosphodiesterase eta-1 OS=Homo sapiens OX=9606 GN=PLCH1
114	☑	☆	Isoform 2 of Neutral amino acid transporter B(0) OS=Homo sapiens OX=9606 GN=SLC1A5
115	☑	☆	Tubulin beta chain OS=Homo sapiens OX=9606 GN=TUBB PE=1 SV=2
116	☑	☆	Isoform 2 of 26S proteasome non-ATPase regulatory subunit 1 OS=Homo sapiens OX=9606 GN=PSMD1
117	☑	☆	Beta-mannosidase OS=Homo sapiens OX=9606 GN=MANBA PE=2 SV=3
118	☑	☆	Protein transport protein Sec31A (Fragment) OS=Homo sapiens OX=9606 GN=SEC31A PE=1 SV=1
119	☑	☆	Prolow-density lipoprotein receptor-related protein 1 OS=Homo sapiens OX=9606 GN=LRP1 PE=1 SV=2
120	☑	☆	Unconventional myosin-If OS=Homo sapiens OX=9606 GN=MYO1F PE=1 SV=3

	Accession Number	Molecular Weight	Protein Grouping Ambiguity	carlosdda_2
	sp|P02461|CO3A1_HUMAN	139 kDa		4,5%
	sp|P11142|HSP7C_HUMAN (+3)	71 kDa		5,9%
	tr|B7Z602|B7Z602_HUMAN	73 kDa		5,0%
	sp|Q02218|ODO1_HUMAN (+1)	116 kDa		2,1%
=...	tr|B3KN37|B3KN37_HUMAN (+1)	68 kDa		4,3%
	sp|Q14315-2|FLNC_HUMAN (+1)	287 kDa	★	2,0%
	sp|P12109|CO6A1_HUMAN (+2)	109 kDa		3,2%
	sp|P52789|HXK2_HUMAN (+3)	102 kDa	★	3,2%
	sp|P45974-2|UBP5_HUMAN (+2)	93 kDa		4,6%
	sp|P26639|SYTC_HUMAN (+3)	83 kDa		6,8%
	sp|Q13200|PSMD2_HUMAN (+2)	100 kDa		5,0%
	sp|P13645|K1C10_HUMAN	59 kDa		5,3%
	sp|P46821|MAP1B_HUMAN (+2)	271 kDa		1,4%
	tr|E9PGZ1|E9PGZ1_HUMAN (+1)	62 kDa		5,8%
	sp|Q92499|DDX1_HUMAN (+1)	82 kDa		2,4%
	sp|Q12797-10|ASPH_HUMAN (+1)	83 kDa		2,5%
	sp|P14735|IDE_HUMAN (+1)	118 kDa		1,5%
	sp|P60709|ACTB_HUMAN (+5)	42 kDa	★	4,5%
	sp|P04083|ANXA1_HUMAN (+1)	39 kDa		3,8%
	sp|Q00341|VIGLN_HUMAN (+2)	141 kDa		2,7%
	sp|Q9NZM1-2|MYOF_HUMAN (+2)	230 kDa		1,9%
	sp|Q14108|SCRB2_HUMAN (+3)	54 kDa		5,4%
	sp|Q16891-3|MIC60_HUMAN (+2)	80 kDa		5,0%
	sp|Q9UDR5|AASS_HUMAN (+1)	102 kDa		3,3%
	tr|A0A2R8Y6G6|A0A2R8Y6G6_HUMAN	47 kDa		3,5%
	sp|P26038|MOES_HUMAN (+1)	68 kDa		2,8%
	sp|Q99798|ACON_HUMAN (+6)	85 kDa		2,1%
	tr|B4DWT9|B4DWT9_HUMAN	61 kDa		2,4%
	sp|Q01995|TAGL_HUMAN (+5)	23 kDa		4,5%
	sp|P33176|KINH_HUMAN (+7)	110 kDa		2,6%
	sp|P40939|ECHA_HUMAN (+5)	83 kDa		3,8%
	sp|Q14194-2|DPYL1_HUMAN (+6)	74 kDa		2,3%
	sp|P35908|K22E_HUMAN	65 kDa	★	1,9%
	sp|P12111-2|CO6A3_HUMAN (+6)	321 kDa		0,30%
	sp|Q13740-2|CD166_HUMAN (+4)	64 kDa		3,2%
	sp|P05106-2|ITB3_HUMAN (+5)	86 kDa		1,5%
	sp|Q12882|DPYD_HUMAN	111 kDa		1,7%
	tr|A0A024R1X6|A0A024R1X6_HUMAN...	30 kDa		4,2%
	sp|Q9NYU2-2|UGGG1_HUMAN (+2)	175 kDa		0,85%
	sp|P11047|LAMC1_HUMAN (+1)	178 kDa		1,8%
	sp|Q6UB35|C1TM_HUMAN (+2)	106 kDa		3,8%
	tr|B4E1J7|B4E1J7_HUMAN	70 kDa		5,5%
	sp|O14980|XPO1_HUMAN	123 kDa		2,8%
	sp|Q8IUD2-2|RB6I2_HUMAN (+7)	114 kDa		1,3%
	sp|Q92598-2|HS105_HUMAN (+8)	92 kDa		1,7%
	sp|P05362|ICAM1_HUMAN (+2)	58 kDa		1,9%
	sp|P0DPH7-2|TBA3C_HUMAN (+18)	46 kDa		4,8%
	sp|P56192|SYMC_HUMAN (+1)	101 kDa		1,2%
	sp|P98194-2|AT2C1_HUMAN (+9)	97 kDa		1,9%
	sp|P19338|NUCL_HUMAN (+4)	77 kDa		2,0%
	tr|D3DVQ1|D3DVQ1_HUMAN	64 kDa		2,1%
	sp|O60313-10|OPA1_HUMAN (+18)	118 kDa		2,8%
	sp|Q4KWH8-2|PLCH1_HUMAN (+2)	185 kDa		0,48%
	sp|Q15758-2|AAAT_HUMAN (+4)	37 kDa		5,9%
	sp|P07437|TBB5_HUMAN (+16)	50 kDa		6,5%
	sp|Q99460-2|PSMD1_HUMAN (+5)	102 kDa		2,8%
	sp|O00462|MANBA_HUMAN (+9)	101 kDa		1,7%
	tr|H0YAB3|H0YAB3_HUMAN	49 kDa		4,5%
	sp|Q07954|LRP1_HUMAN	505 kDa		0,29%
	sp|O00160|MYO1F_HUMAN (+8)	125 kDa		1,5%

Probability Legend:
over 95%
80% to 94%
50% to 79%
20% to 49%
0% to 19%

#	Visible?	Starred?	BioView: Identified Proteins (152)
121	☑	☆	Isoform 2 of Cullin-3 OS=Homo sapiens OX=9606 GN=CUL3
122	☑	☆	Probable leucine--tRNA ligase, mitochondrial OS=Homo sapiens OX=9606 GN=LARS2 PE=1 SV=2
123	☑	☆	Coatomer subunit beta OS=Homo sapiens OX=9606 GN=COPB1 PE=1 SV=3
124	☑	☆	Isoform 2 of Epidermal growth factor receptor substrate 15-like 1 OS=Homo sapiens OX=9606 GN=EPS15L1
125	☑	☆	Isoform 2 of Phosphoglycerate kinase 1 OS=Homo sapiens OX=9606 GN=PGK1
126	☑	☆	Unconventional myosin-Ia OS=Homo sapiens OX=9606 GN=MYO1A PE=1 SV=1
127	☑	☆	Microtubule-associated protein OS=Homo sapiens OX=9606 GN=MAP4 PE=1 SV=1
128	☑	☆	Peptidyl-prolyl cis-trans isomerase FKBP10 OS=Homo sapiens OX=9606 GN=FKBP10 PE=1 SV=1
129	☑	☆	Fatty acid synthase OS=Homo sapiens OX=9606 GN=FASN PE=1 SV=3
130	☑	☆	Isoform 2 of Protein sel-1 homolog 1 OS=Homo sapiens OX=9606 GN=SEL1L
131	☑	☆	Isoform 2 of Sarcoplasmic/endoplasmic reticulum calcium ATPase 2 OS=Homo sapiens OX=9606 GN=ATP2A2
132	☑	☆	Isoform 2 of Glucosidase 2 subunit beta OS=Homo sapiens OX=9606 GN=PRKCSH
133	☑	☆	cDNA FLJ23967 fis, clone HEP16652, highly similar to Golgi apparatus protein 1 OS=Homo sapiens OX=9606 PE=2 SV=1
134	☑	☆	Leucine-rich PPR motif-containing protein, mitochondrial OS=Homo sapiens OX=9606 GN=LRPPRC PE=1 SV=3
135	☑	☆	Isoform 2 of Peroxisomal multifunctional enzyme type 2 OS=Homo sapiens OX=9606 GN=HSD17B4
136	☑	☆	Zyxin OS=Homo sapiens OX=9606 GN=ZYX PE=1 SV=1
137	☑	☆	cDNA FLJ13518 fis, clone PLACE1005799 OS=Homo sapiens OX=9606 PE=2 SV=1
138	☑	☆	cDNA FLJ36998 fis, clone BRACE2007295, highly similar to ALPHA-ADAPTIN A OS=Homo sapiens OX=9606 PE=2 SV=1
139	☑	☆	D-3-phosphoglycerate dehydrogenase OS=Homo sapiens OX=9606 GN=PHGDH PE=1 SV=4
140	☑	☆	Keratin, type I cytoskeletal 9 OS=Homo sapiens OX=9606 GN=KRT9 PE=1 SV=3
141	☑	☆	Pituitary tumor-transforming gene 1 protein-interacting protein OS=Homo sapiens OX=9606 GN=PTTG1IP PE=1 SV=1
142	☑	☆	Actin, cytoplasmic 2 OS=Homo sapiens OX=9606 GN=ACTG1 PE=1 SV=1
143	☑	☆	Integrin beta-5 OS=Homo sapiens OX=9606 GN=ITGB5 PE=1 SV=1
144	☑	☆	Heat shock 70 kDa protein 1A OS=Homo sapiens OX=9606 GN=HSPA1A PE=1 SV=1
145	☑	☆	Cysteine and glycine-rich protein 1 OS=Homo sapiens OX=9606 GN=CSRP1 PE=1 SV=3
146	☑	☆	Nuclease-sensitive element-binding protein 1 OS=Homo sapiens OX=9606 GN=YBX1 PE=1 SV=3
147	☑	☆	cDNA FLJ57106, highly similar to Transferrin receptor protein 1 OS=Homo sapiens OX=9606 PE=2 SV=1
148	☑	☆	cDNA FLJ11291 fis, clone PLACE1009659, highly similar to Nck-associated protein 1 OS=Homo sapiens OX=9606 PE=2 SV=1
149	☑	☆	Isoform 2 of Heterogeneous nuclear ribonucleoprotein D0 OS=Homo sapiens OX=9606 GN=HNRNPD
150	☑	☆	Aldehyde dehydrogenase 18 family member A1 (Fragment) OS=Homo sapiens OX=9606 GN=ALDH18A1 PE=4 SV=1
151	☑	☆	Isoform 11 of Amyloid-beta precursor protein OS=Homo sapiens OX=9606 GN=APP
152	☑	☆	Cystatin-A OS=Homo sapiens OX=9606 GN=CSTA PE=1 SV=1

	Accession Number	Molecular Weight	Protein Grouping Ambiguity	carlosdda_2
	sp|Q13618-2|CUL3_HUMAN (+3)	86 kDa		1,7%
	sp|Q15031|SYLM_HUMAN (+1)	102 kDa		1,8%
	sp|P53618|COPB_HUMAN	107 kDa		1,6%
	sp|Q9UBC2-2|EP15R_HUMAN (+10)	100 kDa		1,4%
	sp|P00558-2|PGK1_HUMAN (+2)	41 kDa		4,6%
	sp|Q9UBC5|MYO1A_HUMAN (+1)	118 kDa		1,6%
	tr|E7EVA0|E7EVA0_HUMAN	245 kDa		0,52%
	sp|Q96AY3|FKB10_HUMAN (+3)	64 kDa		2,6%
	sp|P49327|FAS_HUMAN (+1)	273 kDa		0,68%
	sp|Q9UBV2-2|SE1L1_HUMAN (+2)	34 kDa		7,3%
	sp|P16615-2|AT2A2_HUMAN (+4)	110 kDa		1,5%
	sp|P14314-2|GLU2B_HUMAN (+8)	59 kDa		2,5%
	tr|Q6ZMF1|Q6ZMF1_HUMAN	35 kDa		6,5%
	sp|P42704|LPPRC_HUMAN (+2)	158 kDa		1,2%
	sp|P51659-2|DHB4_HUMAN (+15)	83 kDa		2,5%
	sp|Q15942|ZYX_HUMAN (+3)	61 kDa		3,5%
	tr|Q9H8K1|Q9H8K1_HUMAN	44 kDa		4,3%
	tr|Q8N9K4|Q8N9K4_HUMAN	52 kDa		3,3%
	sp|O43175|SERA_HUMAN (+5)	57 kDa		3,0%
	sp|P35527|K1C9_HUMAN	62 kDa		2,2%
	sp|P53801|PTTG_HUMAN (+3)	20 kDa		10,0%
	sp|P63261|ACTG_HUMAN (+2)	42 kDa	★	4,5%
	sp|P18084|ITB5_HUMAN (+3)	88 kDa		1,9%
	sp|P0DMV8|HS71A_HUMAN (+6)	70 kDa		2,3%
	sp|P21291|CSRP1_HUMAN (+10)	21 kDa		7,8%
	sp|P67809|YBOX1_HUMAN (+4)	36 kDa		5,9%
	tr|B7Z2I6|B7Z2I6_HUMAN (+1)	53 kDa		2,7%
	tr|B3KMK7|B3KMK7_HUMAN	60 kDa		2,7%
	sp|Q14103-2|HNRPD_HUMAN (+4)	36 kDa		4,2%
	tr|A0A2Z4QJH0|A0A2Z4QJH0_HUMAN	7 kDa		21%
	sp|P05067-11|A4_HUMAN (+12)	85 kDa		1,9%
	sp|P01040|CYTA_HUMAN	11 kDa		18%
