# Supplementary material for: Cholinergic-like neurons carrying PSEN1 E280A mutation from familial Alzheimer’s disease reveal intraneuronal sAPPβ fragments accumulation, hyperphosphorylation of TAU, oxidative stress, apoptosis and Ca2+ dysregulation: Therapeutic implications
Source: PLoS One. 2020 May 21;15(5):e0221669. doi: 10.1371/journal.pone.0221669 (PMC7241743; doi:10.1371/journal.pone.0221669)
Supplement: Attachment — Submitted filename: Response to Reviewers R1.docx [file pone.0221669.s008.docx]

We are grateful to editor and reviewers for the time spent and pertinent suggestions intended to improve our manuscript. The response to the specific comments is listed below.

**Journal Requirements:**

When submitting your revision, we need you to address these additional requirements.

1. Please ensure that your manuscript meets PLOS ONE's style requirements, including those for file naming. The PLOS ONE style templates can be found at

http://www .journals.plos.org/plosone/s/file?id=wjVg/PLOSOne_formatting_sample_main_body.pdfand

http://www .journals.plos.org/plosone/s/file?id=ba62/PLOSOne_formatting_sample_title_authors_affiliations.pdf

**Response (R:/)** We have submitted our manuscript as required by PLOS One journal. Please let us know if something is missing to promptly respond.

2. Thank you for letting us know you require assistance with making your Data publicly available. Please note our data availability policy requires authors to make all data underlying the findings described in their manuscript fully available without restriction at the time of publication. When specific legal or ethical requirements prohibit public sharing of a dataset, authors must indicate how researchers may obtain access to the data. (<https://journals.plos.org/plosone/s/data-availability>).

**(R:/)** The authors have made all data underlying the findings described in the manuscript fully available without restriction. Data are submitted as a Supplementary Figures, S1-S5 Figs, Supporting information figures (blots/ gels, S1-S18 I), and supporting information data (Excel format, 1 file). New born de-identified or anonymized genetic data is presented in S1 and S2 Fig according to code TBC #WJMSC-11 – WJMSC-20. The following statement is now included in the manuscript.

**Data availability**

The authors have made all data underlying the findings described in the manuscript fully available without restriction. Data are submitted as a Supplementary Figures, S1-S5 Fig, Supporting information figures (blots/ gels, S1-S18 I), and supporting information data (Excel format, 1 file). New born de-identified or anonymized genetic data is presented in S1 and S2 Fig according to code TBC #WJMSC-11 – WJMSC-20.

If the data is not restricted for public sharing, we strongly recommend depositing your data in a public repository. Please see our Data availability policy for a list of recommended repositories ([https://journals.plos.org/plosone/s/data-availability#](https://journals.plos.org/plosone/s/data-availability)loc-recommended-repositories). In addition, it is also acceptable to upload the data as a Supporting Information file. If the data is restricted from being shared public for ethical or legal reasons, please make the data available upon request and identify the group to which requests should be submitted (e.g., a named data access committee or named ethics committee). The reasons for restrictions on public data deposition must also be specified. Note that it is not acceptable for an author to be the sole named individual responsible for ensuring data access. <https://journals.plos.org/plosone/s/dataavailability#loc-acceptable-data-sharing-methods>).

**R:/** The data is not restricted for public sharing. As suggested, we have upload the data as a Supplementary Figures, S1-S5 Fig, Supporting information figures (blots/ gels, S1-S18 I), and supporting information data (Excel format, 1 file). New born de-identified or anonymized genetic data is presented in S1 and S2 Fig according to code TBC #WJMSC-11 – WJMSC-20.

3. PLOS ONE now requires that authors provide the original uncropped and unadjusted images underlying all blot or gel results reported in a submission’s figures or Supporting Information files. This policy and the journal’s other requirements for blot/gel reporting and figure preparation are described in detail at <https://journals.plos.org/plosone/s/figures#loc-blotand-gel-reporting-requirements> and https://journals.plos.org/plosone/s/figures#loc-preparing-figures-from-image-files.

When you submit your revised manuscript, please ensure that your figures adhere fully to these guidelines and provide the original underlying images for all blot or gel data reported in your submission. See the following link for instructions on providing the original image data: <https://journals.plos.org/plosone/s/figures#loc-original-images-for-blots-and-gels>. In your cover letter, please note whether your blot/gel image data are in Supporting Information or posted at a public data repository, provide the repository URL if relevant, and provide specific details as to which raw blot/gel images, if any, are not available. Email us at plosone@plos.orgif you have any questions.

**(R:/)** We are providing the original uncropped and unadjusted images underlying all blots or gels reported as a Supporting Information (S1-S18 I) and Supporting Information data in Excel format (one file).

4. We note that you have included the phrase “data not shown” in your manuscript. Unfortunately, this does not meet our data sharing requirements. PLOS does not permit references to inaccessible data. We require that authors provide all relevant data within the paper, Supporting Information files, or in an acceptable, public repository. Please add a citation to support this phrase or upload the data that corresponds with these findings to a stable repository (such as Figs hare or Dryad) and provide and URLs, DOIs, or accession numbers that may be used to access these data. Or, if the data are not a core part of the research being presented in your study, we ask that you remove the phrase that refers to these data.

**(R:/)** The phrase “data not shown” has been removed from the manuscript.

**Editor Dr. Firas Kobeissy’s Comments**

I would request that the authors perform a mass spec analysis of the proposed oligomeric Abeta 42 in Figure 3A (the band should provide some peptides of Abeta proteins).

**(R:/)** This is a most pertinent requirement. As suggested by editor, the proposed oligomeric Abeta 42 was analyzed by LC-MS/MS (please see Supporting Material (S5A, B, and C Fig), Results and Discussion sections). Surprisingly, “To confirm the identity of the intracellular APP/ Aβ_42_ aggregates displayed in the Western blot (**Fig.** **3A**, *arrowhead*), we used LC-MS/MS technique. The LC-MS/MS analysis identified two peptides ^364^LPTTAASTPDAVDK^377^ (**S5A Fig**) and ^430^AVIQHFQEK^438^ (**S5B Fig**) that showed high homology with APP fragments (e.g., APP714, APP733, APP751, APP752, **S5C Fig**), hereafter sAPPβf. Strikingly, no Aβ42 fragment was identified in the interrogated gel region”. (pag. 25, line 15-20). We therefore concluded that “Taken together these observations suggest that sAPP fragments rather than Aβ42 are accumulating early in the PSEN1 E280A cholinergic neurons” (pag. 34, line 19-21). Furthermore “Since the PSEN1 E280A mutation might represent a typical trans-dominant negative mutation on γ-secretase (Heilig et al., 2013; Zhou et al., 2017), we do not discard the possibility that APP-derived intracellular Aβ42 aggregates might be a late event in the process of neurodegeneration (Takahashi et al., 2017; Wirths et al., 2004; LaFerla et al., 2007)” (pag. 35, line 13-15).

Reviewers' comments

**Reviewer's Responses to Questions**

**Comments to the Author**

1. Is the manuscript technically sounds, and do the data support the conclusions?

The manuscript must describe a technically sound piece of scientific research with data that supports the conclusions. Experiments must have been conducted rigorously, with appropriate controls, replication, and sample sizes. The conclusions must be drawn appropriately based on the data presented.

Reviewer #1: Yes

Reviewer #2: Partly

2. Has the statistical analysis been performed appropriately and rigorously?

Reviewer #1: I Don't Know **(R:/)** On pag. 19, in original manuscript, we provided a detailed description of the statistical analysis of the data (Data analysis section) including Ref. [38].

Reviewer #2: Yes

3. Have the authors made all data underlying the findings in their manuscript fully available?

The PLOS Data policy requires authors to make all data underlying the findings described in their manuscript fully available without restriction, with rare exception (please refer to the Data Availability Statement in the manuscript PDF file). The data should be provided as part of the manuscript or its supporting information, or deposited to a public repository. For example, in addition to summary statistics, the data points behind means, medians and variance measures should be available. If there are restrictions on publicly sharing data—e.g. participant privacy or use of data from a third party—those must be specified.

Reviewer #1: No **(R:/)** A data availability statement is now included in the manuscript (pag 42), supporting information figures and supporting information data (in excel format) are now provided.

Reviewer #2: Yes

4. Is the manuscript presented in an intelligible fashion and written in standard English?

PLOS ONE does not copyedit accepted manuscripts, so the language in submitted articles must be clear, correct, and unambiguous. Any typographical or grammatical errors should be corrected at revision, so please note any specific errors here.

Reviewer #1: Yes

Reviewer #2: Yes

**5. Review Comments to the Author**

Please use the space provided to explain your answers to the questions above. You may also include additional comments for the author, including concerns about dual publication, research ethics, or publication ethics. (Please upload your review as an attachment if it exceeds 20,000 characters)

Reviewer #1: This manuscript described a new and effective way of cell culture model that provides novel opportunities to study the mechanisms of AD on cellular level, which potentially will become a good model for therapeutic study. In the future, more studies should be done using this model to compare different mutations. Better including siblings as controls to limit the genetic background variation. If this method is widely adopted, a large MSCs bank should be setup for the study of all kinds of human diseases. The data is very impressive. There are a few small things I would like to address:

1. You collected 10 samples. Only 1 of them has the mutation. You also only randomly picked 1 out of 9 as the WT control. Did you ever test any of the other 8? If yes, could you show the data? Any of the other 8 have ApoE 3/4, which could be a better control. In fact, you should use more than one WT control, since you have enough resource. If possible, could you add another one, preferred ApoE 3/4, to the data?

**(R:/)** We did not test all samples but only for the selected one described in the present work (i.e. wild type and mutant PSEN1 E280A). Based on the fact that the mutation PSEN1 E280A is 100% penetrant in those individuals bearing the mutation, we just follow the following selection process. (Pag.7 lines 5-11) “The PSEN1 E280A mutation was detected by PCR using mismatch primers and digestion of the products with Bsm1 [28]. Digested products were separated on a 3% agarose gel. According to different mobility electrophoretic patterns, samples were classified as wild-type (WT) or mutant PSEN1 E280A when compared to PSEN1 E280A carrier (positive case NeuroBank (NB) code #18233) or wild type PSEN1 genotype (NB code#18574). The TBC# WJMSC-12 (female) sample was identified for PSEN1 E280A mutation (S1 Fig). For comparative purposes, we pre-selected female WT PSEN1 samples (TBC# WJMSC-11, -15, -17), and TBC# WJMSC-11 WT PSEN1 was randomly selected for further experiments”. As required by referee, genotype of 10 samples is now included (S1 Fig).

It is well known that the strongest known genetic risk factor for the development of late-onset Alzheimer disease is inheritance of the apolipoprotein (APO) E4 allele. Indeed, lipoprotein transport system is mediated by high density lipoprotein-sized. The APO E-containing lipoproteins are synthesized and secreted by glial cells (primarily astrocytes). The astrocyte-derived APO E-containing lipoproteins can bind to, and be internalized by, receptors of the low density lipoprotein receptor superfamily that are located on the surface of neurons. We therefore think that the APOE profile of the wild type APOE 3/3 and APOE 3/4 ChLNs are not critical for the analysis of early onset FAD cholinergic neurons as shown in the present investigation. We consider that the APOE might be informative for future studies where neurons and astrocytes interaction would be investigated. The APOE profile of samples (n=10) are now included (S2 Fig).

2. Since you only tripicate of the experiment, could you run all the 3 repeats on the same gels? Please use that the replace the western blot pictures on figure 2, 3 and 5.

**(R:/)** As suggested by the reviewer, all the 3 repeats of the experiments are now run on the same gels on Fig 2, 3 and 5. Please note that we now included Fig 7 as for figures 2,3,5.

3. In figure 3, we can see pretty high levels of Abeta42 on Figure A, but almost 0 on figure J. How to explain it?

**(R:/)** The referee remark is right. We think the difference between Fig 3A and Fig J is technical. While Western blot (WB) uses cell extract for protein analysis, IF analysis of protein is at single-cell level. Therefore, Western blot is more sensible than IF. Alternatively, the antibody which recognized c-terminal Abeta (AB5078P) may not be optimal. To get solved this problem, as suggested by referee 1 and 2, we have used the antibody 6E10. Noticeably, the use of this antibody now corrects for this. The results are now presented in Fig. 3A and 3J with similar observations for both WB and IF. However, as LC-MS/MS analysis proved, no Aβ42 but rather sAPPβ fragments were identified in the WB-HMW band.

4. The Figure 3E and F showed nothing, comparing to H and I, which is not consistent with western blot at figure A.

**(R:/)** As noted in item 3, this discrepancy has been solved with the use of antibody 6E10.

5. At figure A, you didn't detect any monomer of Abeta42 at all, which is not consistent from our experience. In order to test whether your anitbody is specific enough for Abeta, could you repeat the same figure using another Abeta or APP antibody, such as 6E10?

**(R:/)** As suggested by the referee, we have repeated the detection of synthetic Abeta 42 with antibody 6E10. As shown in blot, and similar to c-terminus Abeta antibody AB5078P, no Abeta42 monomers were detected**.**

6. The quality of the western blot figure of Figure 3A on OxDJ-1 Cys106 is too poor. Do you have a better picture? Anyway, you should rerun the whole gel with all 3 repeat on the same gel. You can buy 18 well gel from Bio-Rad.

**(R:/)** As suggested by the reviewer, the quality of the WB has been improved. We have rerun the whole gel with all 3 repeat on the same gel. Please note however that since there were no differences between day 2 and 4, we selected 0 and 4 day to rerun the gels. Also note that 15 well gel but not 18 well gel was selected for technical easiness and availability of the complete set of apparatus involving protein electrophoresis apparatus plus + Western blotting apparatus, among other reagents. Please note that similar data at day 0 and 4 were obtained as submitted originally.

7. You talked about Ca2+ flux at many places in the manuscript, but there is no data to show it. Did you forget to put the figures in?

**(R:/)** Fig 6 was successfully submitted in the original ms wherein the Ca2+ flux experiments were shown. Please see it again in the present ms version.

8. At the method part of the Isolation and Expansion of hWJ-MSCs, 2000 rpm, using x g is better, since people will have different centrifuge than yours.

**(R:/)** As suggested, rpm has been changed to x *g* through material and method text.

9. At the method part of western blot, could you list the dilution of the primary antibodies so that other labs can follow?

**(R:/)** The dilution of the primary antibodies appeared in the original manuscript (pag. 11 and 12).

10. On page 16, did you use 400x objective of the microscope. Usually 100x is the highest amplification of the objective. **(R:/)** This error has been corrected. It is 100x as noted**.**

Reviewer #2: The paper by Soto-Mercado examines the production and oligomerization of Abeta in induced cholinergiec neurons derived from umbilical cord mesenchymal stem cells. The authors report that they initially began the study with 10 different umbilical cord samples of which only 1 was a gene carrier for the PSEN1 E280A mutation. The study then focuses on cultures derived from one of the 9 samples lacking a mutation for comparison to the one culture with the E280A mutation. A brief explanation on how the one control sample was chosen would be useful. Was it chosen at random, or for some other property?

**(R:/)** As suggested by referee 1 and 2, a brief explanation as to how samples were chosen is now included (pag. 7, lines 5-11). Based on the fact that the mutation PSEN1 E280A is 100% penetrant in those individuals bearing the mutation, we just follow the following selection process. “The PSEN1 E280A mutation was detected by PCR using mismatch primers and digestion of the products with Bsm1 [28]. Digested products were separated on a 3% agarose gel. According to different mobility electrophoretic patterns, samples were classified as wild-type (WT) or mutant PSEN1 E280A when compared to PSEN1 E280A carrier (positive case NeuroBank (NB) code #18233) or wild type PSEN1 genotype (NB code#18574). The TBC# WJMSC-12 (female) sample was identified for PSEN1 E280A mutation (S1 Fig). For comparative purposes, we pre-selected female WT PSEN1 samples (TBC# WJMSC-11, -15, -17), and TBC# WJMSC-11 WT PSEN1 was randomly selected for further experiments”.

It is a weakness of the study that other cultures from non-mutant carriers were not characterized to provide a view of the variability of the characteristics of these cultures. As such, the study is really a case-study of one isolated MSC culture derived from one individual compare to a single individual without the mutation.

**(R:/)** The reviewer is right, this is a case-study of one isolated MSC culture derived from one individual compare to a single individual without the mutation. As mentioned before, since the mutation is 100% penetrant, we consider that one single control might suffice for the purpose of the present study. For the sake of clarity, the sentence “Despite the fact that the mutation PSEN1 E280A is 100% penetrant in those individuals bearing such mutation, and given that our findings are based on ChLNs obtained from a single PSEN1 E280A MSCs, the conclusions from such analyses require verification through additional studies with larger sample size ” is now included (pag 40, line 10-13).

In my opinion, the conclusions that the authors draw from the study of these cultures are over-stated.

**(R:/)** Given the observation that no Aβ42 but rather sAPPβ fragments were identified in the WB-HMW band by LC-MS/MS analysis, we think that the conclusions are now adjusted to observations. Furthermore, for the sake of clarity, the sentence “Despite the fact that the mutation PSEN1 E280A is 100% penetrant in those individuals bearing such mutation, and given that our findings are based on ChLNs obtained from a single PSEN1 E280A MSCs, the conclusions from such analyses require verification through additional studies with larger sample size ” (pag. 42, line 10-13).

The authors state that the major implication of their study is that they find an important role for intracellular Abeta in cholinergic neurons expressing mutant SOD1. **(R:/)** in mutant PSEN1 not in SOD1. Given the emphasis on this conclusion, the authors need to provide more evidence that the bands that are detected as oligomeric Abeta 42 in Figure 3A are indeed Abeta. **(R:/)** This is most pertinent remark. As suggested by the reviewer and Editor, we have now repeated all experiments with antibody 6E10, and submitted band (aggregate Abeta) to mass spectrometry analysis (please see results section). Surprisingly, “To confirm the identity of the intracellular APP/ Aβ_42_ aggregates displayed in the Western blot (**Fig.** **3A**, *arrowhead*), we used LC-MS/MS technique. The LC-MS/MS analysis identified two peptides ^364^LPTTAASTPDAVDK^377^ (**S5A Fig**) and ^430^AVIQHFQEK^438^ (**S5B Fig**) that showed high homology with APP fragments (e.g., APP714, APP733, APP751, APP752, **S5C Fig**), hereafter sAPPβf. Strikingly, no Aβ42 fragment was identified in the interrogated gel region”. (pag. 25, line 15-20). We therefore concluded that “Taken together these observations suggest that sAPP fragments rather than Aβ42 are accumulating early in the PSEN1 E280A cholinergic neurons” (pag. 34, line 19-21). Furthermore “Since the PSEN1 E280A mutation might represent a typical trans-dominant negative mutation on γ-secretase (Heilig et al., 2013; Zhou et al., 2017), we do not discard the possibility that APP-derived intracellular Aβ42 aggregates might be a late event in the process of neurodegeneration (Takahashi et al., 2017; Wirths et al., 2004; LaFerla et al., 2007)” (pag. 35, line 13-15).

Although the antibody appears to be specific to the C-terminus of Abeta42, the authors need to provide additional evidence that the immunoreactive bands in Fig. 3A are really oligomeric Abeta 42. For example, can the authors demonstrate that the same bands are recognized by an antibody that recognizes the N-terminus of Abeta such as clone 1E8 (MABN639 from Millipore/Sigma).

**(R:/)** As suggested by the reviewer, we have now repeated all experiments with antibody 6E10 and have found consistently that N-terminus antibody (6E10) provides similar results and conclusions as antibody that recognized c-terminus of Abeta. Additionally, we included LC-MS/MS analysis. We conclude that antibody 6E10 recognized high molecular aggregates of sAPPβ.

Additionally, given the intensity of antibody immunoreactivity in western blots of WT cells at 4 hours, it is surprising that the immunostaining of these cells in panels D-F is so weak. I would again like to see staining with an N-terminal antibody Abeta antibody.

**(R:/)** As mentioned, this immunostaining weakness has now been resolved with the use of (6E10).

6. PLOS authors have the option to publish the peer review history of their article (what does this mean?). If published, this will include your full peer review and any attached files.

If you choose “no”, your identity will remain anonymous but your review may still be made public.

Do you want your identity to be public for this peer review? For information about this choice, including consent withdrawal, please see our Privacy Policy.

Reviewer #1: No

Reviewer #2: No
