# Supplementary material for: Cholinergic-like neurons carrying PSEN1 E280A mutation from familial Alzheimer’s disease reveal intraneuronal sAPPβ fragments accumulation, hyperphosphorylation of TAU, oxidative stress, apoptosis and Ca2+ dysregulation: Therapeutic implications
Source: PLoS One. 2020 May 21;15(5):e0221669. doi: 10.1371/journal.pone.0221669 (PMC7241743; doi:10.1371/journal.pone.0221669)
Supplement: Attachment — Submitted filename: Response to Reviewers R2.docx [file pone.0221669.s009.docx]

We are grateful to editor and reviewers for the time spent and pertinent suggestions intended to improve our manuscript. The response (**R/**) to the specific comments is listed below.

Dear Authors,

thank you for the submission and we truly appreciate the hard work in answering the previous concerns. There are some minor comments related to the result sections and data interpretation pertaining to aim APP and Abeta 42 levels reporting in the figures.

Please address these concerns.

Thank you

**Response (R/).** Minor comments have fully been addressed. Thank you.

Reviewers' comments:

Reviewer's Responses to Questions

Comments to the Author

1. If the authors have adequately addressed your comments raised in a previous round of review and you feel that this manuscript is now acceptable for publication, you may indicate that here to bypass the “Comments to the Author” section, enter your conflict of interest statement in the “Confidential to Editor” section, and submit your "Accept" recommendation.

Reviewer #1: All comments have been addressed

Reviewer #2: (No Response)

2. Is the manuscript technically sound, and do the data support the conclusions?

The manuscript must describe a technically sound piece of scientific research with data that supports the conclusions. Experiments must have been conducted rigorously, with appropriate controls, replication, and sample sizes. The conclusions must be drawn appropriately based on the data presented.

Reviewer #1: Yes

Reviewer #2: Partly

3. Has the statistical analysis been performed appropriately and rigorously?

Reviewer #1: Yes

Reviewer #2: I Don't Know

4. Have the authors made all data underlying the findings in their manuscript fully available?

The PLOS Data policy requires authors to make all data underlying the findings described in their manuscript fully available without restriction, with rare exception (please refer to the Data Availability Statement in the manuscript PDF file). The data should be provided as part of the manuscript or its supporting information, or deposited to a public repository. For example, in addition to summary statistics, the data points behind means, medians and variance measures should be available. If there are restrictions on publicly sharing data—e.g. participant privacy or use of data from a third party—those must be specified.

Reviewer #1: Yes

Reviewer #2: Yes

5. Is the manuscript presented in an intelligible fashion and written in standard English?

PLOS ONE does not copyedit accepted manuscripts, so the language in submitted articles must be clear, correct, and unambiguous. Any typographical or grammatical errors should be corrected at revision, so please note any specific errors here.

Reviewer #1: Yes

Reviewer #2: Yes

6. Review Comments to the Author

Please use the space provided to explain your answers to the questions above. You may also include additional comments for the author, including concerns about dual publication, research ethics, or publication ethics. (Please upload your review as an attachment if it exceeds 20,000 characters)

**Reviewer #1:** This manuscript described a new and effective way of cell culture model that provides novel opportunities to study the mechanisms of AD on cellular level, which potentially will become a good model for therapeutic study. The data is very impressive. The corrected version already addressed all my comments and included a lot of extra effort. It is a good study.

**Reviewer #2:** The authors have largely addressed my original criticisms. However, the new data raise a new question that requires clarification. The authors indicate that the induced neurons with the PS1 mutation have increased levels of an APP fragment. In Figure 3, the authors have 2 time points post differentiation – 0 day and 4 days. I really see also most no change in the levels of most of what is measured over the 4 days. In Figure S3, the authors show that MSCs in regular medium with the PS1 mutation have no detectable levels of the intracellular APP fragments. I am struggling to understand why the 0 day for the mutant cells in the differentiation paradigm do not match the undifferentiated MSCs? A similar question applies to the oxidized DJ-1 data.

**R/** For the sake of clarity, the sentence “Western blot measurements and immunofluorescence analysis revealed that both WT and PSEN1 E280A MSCs displayed undetectable levels of intracellular APP / Aβ42 and oxidized DJ-1 at days 0, 2 and 4 post differentiation (S3A-J Fig)” has been changed to “After 7 days of culture in MCm, MSCs were left in RCm for 0, 2 and 4 additional days. Western blot measurements and immunofluorescence analysis revealed that both WT and PSEN1 E280A MSCs displayed undetectable levels of intracellular APP / Aβ42 and oxidized DJ-1 at days 0, 2 and 4 (S3A-J Fig)” (p24, L9-10). Briefly, MSCs were culture in non-differentiation media (i.e., in MCm) and differentiation media (i.e., Ch-N-Rm) for 7 days. After this time, both non-differentiated (i.e., MSCs) and differentiated cells (i.e., ChLNs) were exposed to RCm for 0, 2, and 4 additional days.

The only proteins showing an obvious temporal change in levels are Caspase 3 and p53. In the Discussion, the authors state that day 0 is after 7 days of differentiation – in which case day is really day 7 and day 4 is really day 11. Please be precise in your descriptions.

**R/** As rightly inferred by the referee, day 0 corresponded to 7 days of differentiation. For clarity, the sentence “Since Ch-N-Rm contains several factors that might interfere with the experiment interpretation and measurements, WT PSEN1 and PSEN1 E280A ChLNs (obtained after 7 days in Ch-N-Rm) were left in regular culture medium (RCm) for 0, 2 or 4 additional days, hereafter 0, 2, or 4 days of post transdifferentiation” is now added (p 10, L22-23; p11 L1-2). Please also note that, for most of the procedures, the sentence “ChLNs were left in regular medium (RCm) for 0, 2 and 4 days” was included in the original manuscript.

The abstract mentions increased secretion of Abeta 42 by the mutant cells, but I did not see any data to support this conclusion.

**R/** Please note that increased secretion of Abeta 42 by the mutant cells was described in the original version of this work in Materials and methods section (item Measurement of Aβ1-42 peptide in culture medium (p17) and data were shown in Fig. 6A.

If the APP immunostaining data is accurate, the authors can only associate a change in APP processing with other toxic changes. There is no data to establish that the APP fragment detected is causative of any other characteristic of these cells. siRNA knockdown of APP would help establish causation.

**R/** As mentioned in discussion section “Whether intracellular APP fragments build up because full length APP processing is altered and consequently some APP fragments remain intracellular leading to cell death require further investigation. However, recent data support this view. Indeed, it has been shown that hAPP overexpression causes Aβ-independent neuronal death in olfactory sensory neurons via intrinsic apoptosis pathway [5, 6]” (p35, L2-6). Additionally, when comparing wild type versus mutant PSEN1 E280A ChLNs, all deleterious effects observed (e.g., OS, tau-phosphorylation, etc.) appeared only in mutant ChLNs simultaneously with appearance of sAPPβf, identified by LC-MS/MS. Taken together these observations suggest that sAPPβf is causative of herein described observations. We thank the reviewer for the suggestion to siRNA knockdown of APP. However, this procedure is out of scope of the present investigation, and will be explored in a near future.

It is not clear to me how the authors can conclude that this cell model is a valid model of AD. The data suggest that soon after differentiation, neurons with mutant PS1 would be induced to die, which clearly does not happen in these individuals.

**R/** For the sake of clarity, the sentence “…are a valid model of FAD and…” in abstract has been deleted. Effectively, further investigation in newborn brains bearing the PSEN1 E280A mutation is needed to fully establish whether or not this mutation accelerate neural cell death in these individuals. However, recent data suggest that “Studies from this cohort suggest that structural and functional brain abnormalities - such as cortical thinning and hyperactivation in memory networks - as well as differences in biofluid and in vivo measurements of Alzheimer's-related pathological proteins distinguish Presenilin1 E280A mutation carriers from non-carriers as early as childhood, or approximately three decades before the median age of onset of clinical symptoms” (in ref. [13] Fuller JT, et al. Biological and Cognitive Markers of Presenilin1 E280A Autosomal Dominant Alzheimer's Disease: A Comprehensive Review of the Colombian Kindred. J Prev Alzheimers Dis 2019;6(2):112-120).

7. PLOS authors have the option to publish the peer review history of their article (what does this mean?). If published, this will include your full peer review and any attached files.

If you choose “no”, your identity will remain anonymous but your review may still be made public.

Do you want your identity to be public for this peer review? For information about this choice, including consent withdrawal, please see our Privacy Policy.

Reviewer #1: No

Reviewer #2: No
